# Optical Fibre-Based Pulse Oximetry Sensor with Contact Force Detection

**DOI:** 10.3390/s18113632

**Published:** 2018-10-26

**Authors:** Chong Liu, Ricardo Correia, Hattan Khaled Ballaji, Serhiy Korposh, Barrie R. Hayes-Gill, Stephen P. Morgan

**Affiliations:** Optics and Photonics Group, Faculty of Engineering, University Park, Nottingham NG7 2RD, UK; chong.liu@nottingham.ac.uk (C.L.); Ricardo.GoncalvesCorreia@nottingham.ac.uk (R.C.); hattan.ballaji@nottingham.ac.uk (H.K.B.); S.Korposh@nottingham.ac.uk (S.K.); barrie.hayes-gill@nottingham.ac.uk (B.R.H.-G.)

**Keywords:** pulse oximetry, plastic optical fibre, oxygen saturation, photoplethysmography, fibre Bragg grating, contact pressure, signal quality index

## Abstract

A novel optical sensor probe combining monitoring of blood oxygen saturation (S_p_O_2_) with contact pressure is presented. This is beneficial as contact pressure is known to affect S_p_O_2_ measurement. The sensor consists of three plastic optical fibres (POF) used to deliver and collect light for pulse oximetry, and a fibre Bragg grating (FBG) sensor to measure contact pressure. All optical fibres are housed in a biocompatible epoxy patch which serves two purposes: (i) to reduce motion artefacts in the photoplethysmogram (PPG), and (ii) to transduce transverse loading into an axial strain in the FBG. Test results show that using a combination of pressure measuring FBG with a reference FBG, reliable results are possible with low hysteresis which are relatively immune to the effects of temperature. The sensor is used to measure the S_p_O_2_ of ten volunteers under different contact pressures with perfusion and skewness indices applied to assess the quality of the PPG. The study revealed that the contact force ranging from 5 to 15 kPa provides errors of <2%. The combined probe has the potential to improve the reliability of reflectance oximeters. In particular, in wearable technology, the probe should find use in optimising the fitting of garments incorporating this technology.

## 1. Introduction

The blood oxygen saturation level (S_p_O_2_) indicates the percentage of oxygenated haemoglobin molecules in the arterial blood, which has been identified as an indicator of risk in chronic disease of the circulatory and respiratory system and is required to be continuously monitored during for example anaesthesia [1]. Pulse oximetry is a non-invasive method to detect S_p_O_2_ which was first introduced in 1983 and accepted as a standard procedure in administrating general anaesthetic in the US in 1987 [2]. The S_p_O_2_ measured by pulse oximetry is defined as the ratio between the oxygenated haemoglobin (HbO_2_) and the total haemoglobin (HbO_2_ + Hb):

(1) $$S_{P}O_{2}=\frac{{\mathrm{HbO}}_{2}}{{\mathrm{HbO}}_{2}+\mathrm{Hb}}\times 100\%$$

Since the absorption spectra of HbO_2_ and Hb are different, pulse oximetry uses photoplethysmography (PPG) at two different wavelengths (usually red and near-infrared) to obtain the S_p_O_2_ value [3,4]. The PPG signal is the intensity of the light penetrating or reflected by body tissues, which consists of a small pulsatile component (AC) and a large static component (DC). The light absorption of arterial blood causes the pulsatile component in PPG signals whilst the light absorption of steady component (due to venous blood, bone, skin, hair and tissue) gives rise to the DC component [5]. Pulse oximetry calculates S_p_O_2_ from the absorbance ratio (R) which is derived from the ratios of pulsatile components of PPG signals (Iac) to static components of PPG signals (Idc).

(2) $$R=\frac{I_{\mathrm{ac},\mathrm{red}}/I_{\mathrm{dc},\mathrm{red}}}{I_{\mathrm{ac},\mathrm{infrared}}/I_{\mathrm{dc},\mathrm{infrared}}}$$

Pulse oximetry can be performed in transmission and reflectance geometries [6]. Transmission mode is limited to extremities such as the finger, toe or earlobe whereas reflectance mode provides greater flexibility of body site. Unobtrusive wearable reflectance pulse oximetry has attracted a lot of research attention [7] with optical fibres providing a convenient method of delivering light to and from the body [8,9]. This is useful in magnetic resonance imaging (MRI) where metallic parts should be avoided [10]. In recent years, there has also been interest in integrating optical fibres into fabrics to make wearable photonic textiles for comfortable and continuous monitoring of the subjects’ S_p_O_2_ [7,11,12].

However, there are challenges in using photonic textiles for pulse oximetry. Inefficient light coupling at the tissue interface means that many optical fibres are required [13,14]. Motion artefacts also occur when the fibres move relative to the tissue surface and so the sensor needs to be in contact with the skin which requires tight fitting garments or straps making it impractical for long-term use. It is also known that the contact force between tissue and sensor will affect the accuracy of S_p_O_2_ values in both transmission and reflectance modes [15,16,17]. Insufficient contact pressure can cause a weak PPG signal whilst too high pressure will block the blood circulation and deform the PPG [18]. A range of contact force exists to generate optimal PPG signals with salient pulsatile components [15,17,19].

It would therefore be advantageous to develop an optical fibre sensor that could monitor both the PPG and the contact pressure. This would enable indication of when the probe is in contact with the tissue and when it is in the appropriate contact pressure range to enable reliable S_p_O_2_ measurement. An optical fibre sensing method of monitoring contact pressure can be obtained using a Fibre Bragg Grating (FBG). An FBG consists of a periodic variation of the refractive index of the core [20]. For a particular effective refractive index (n_e_) modulation, light will be reflected by the grating at a specific wavelength (the Bragg wavelength λ_B_) which is dependent on the grating period (Λ).

(3) $$\lambda_{B}=2\times n_{e}\times \Lambda$$

The variation of the grating period and the refractive index caused by vertical transverse loading, axial strain or temperature will shift the Bragg wavelength. However, the bare FBG sensor is not very sensitive to the vertical transverse load and in order to increase its sensitivity the bare FBG sensor can be encased in an epoxy-based UV-cured rectangular block or patch [21]. In this case, the vertical transverse load, the result of the applied pressure is transduced into a horizontal axial strain and hence small transverse pressure variations (~100 Pa) can cause a measurable Bragg wavelength shift.

This paper demonstrates a novel optical fibre sensor probe for simultaneously monitoring the PPG and contact pressure in order to provide reliable S_p_O_2_ monitoring. The probe is encased in an epoxy patch which serves to fix the S_p_O_2_ measurement fibres in place and to transduce a transverse load into an axial strain at the FBG. The next section describes the sensing system and the experiments conducted. Calibration and human volunteer results and discussion are shown Section 3 followed by conclusions in Section 4.

## 2. Materials and Methodology

### 2.1. Probe Design

**Figure 1:**
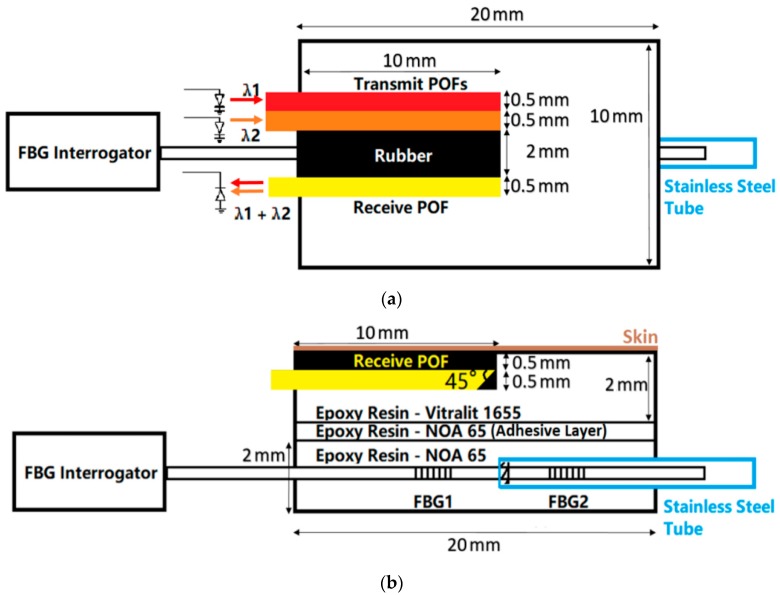
(**a**) Plan view of the probe. Red and orange frames represent transmit POFs connected to two LEDs (660 nm and 850 nm). The yellow frame represents the receiver POF connected to the photo-diode. (**b**) Side view of the probe. The probe consists of two epoxy rectangular patches which are connected using epoxy resin. The top patch is the pulse oximeter whilst the bottom is the FBG pressure sensor. Two FBGs are fabricated in the core of the silica fibre which are connected to the FBG interrogator (see Section 2.2). The brown line represents the skin surface where the POFs are located.

Figure 1 shows the probe design. The designed probe is composed of two patches which are a pulse oximeter (the top patch) and an FBG pressure sensor (the bottom patch). Two patches are glued together using epoxy resin (NOA-65, Norland Products, NJ, USA). The pulse oximeter consists of three 500 µm diameter POFs (DB-500, Asahi Kasei, Tokyo, Japan) to deliver/receive light from/to the opto-electronic system. The end of each POF embedded in the epoxy patch is 45° cleaved using a sharp blade to increase the light reflecting from the side of the angled optical fibre, which allows for sensing away from the axis of the fibre [22]. This has been previously demonstrated using micro-prisms to be an efficient method of delivering light to tissue for pulse oximetry [23]. Although a 45° cleaved fibre was reported to be able to achieve high optical coupling efficiency in low-height and low-cost optical interconnect modules [24], to our knowledge, this has not been widely adopted to increase the coupling efficiency of POF pulse oximeters.

The bottom substance of the pulse oximeter patch is made of epoxy resin (Vitralit 1655, Panacol-Elosol GmbH, Steinbach, Germany) which is a biocompatible material. Exposing to UV light with a maximum absorption within the wavelength range of 350–380 nm for 15 min, the epoxy resin is cured in a mould into a 20 × 10 × 2 mm cube. There is one 3.5 × 10 × 1 mm slot on the top surface of the epoxy cube generated by polishing. Three POFs with 45° cleaved and polished ends are set into this slot and then fixed by filling and curing the epoxy resin. Black rubber (2 mm thick) on the surface of the epoxy patch between transmit and receive POFs is present to prevent light passing directly from the source to the detector as this would affect Idc and hence the S_p_O_2_ value (Equation (2)).

For contact pressure sensing, two FBGs, separated by 6 mm are written into a 125 µm diameter photosensitive silica optical fibre (PS1250, Fibercore Ltd., Southampton, UK) using a UV inscription method previously described [21]. Encasing an FBG in epoxy transduces a transverse load into an axial strain. However, temperature changes will cause shifts to the Bragg wavelength and therefore a reference FBG is required to compensate. The reference FBG is shielded in a stainless-steel tube (outer diameter: 0.56 mm, inner diameter: 0.305 mm, Coopers Needle Works Ltd., Birmingham, UK) to make it sensitive to only temperature. Figure 1 shows the schematic of the FBG pressure sensor. FBG1 is covered by the epoxy rectangular cube to measure the pressure whilst FBG2 is protected by a stainless-steel tube enabling measurement of temperature and insensitivity to pressure. The patch is made of epoxy resin NOA-65 which has lower Young’s modulus (137.9 MPa) and cured in the same mould as the pulse oximeter patch into a 20 × 10 × 2 mm after exposure to UV light.

### 2.2. Opto-Electronic System

**Figure 2:**
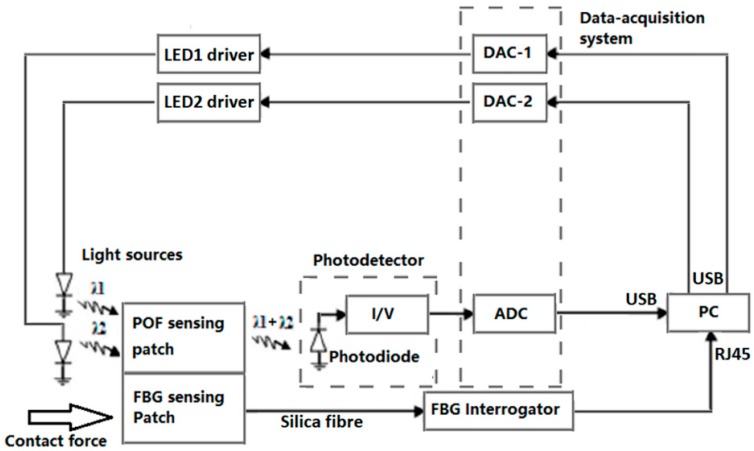
Opto-electronics system for the designed sensor. Light sources are two different wavelength Thorlabs fibre-coupled LEDs (660 nm and 850 nm). The photodetector is a Thorlabs PDA36AEC. The data-acquisition system is a National Instruments myDAQ at 16 bits and 20 kHz sampling frequency. The FBG interrogator is a Smart Fibres SmartScan FBG interrogator with 0.8 pm resolution. (I/V—current to voltage converter, DAC—digital-to-analogue converter, ADC—analogue-to-digital converter and PC—personal computer).

Figure 2 shows a block diagram of the opto-electronics system. The reflectance pulse oximetry sensor part consists of two LEDs operating at different wavelength: one at λ = 660 nm (fibre-coupled LED M00408143, Thorlabs, NJ, USA); and the other at λ = 850 nm (fibre-coupled LED M00290109, Thorlabs, NJ, USA). Two LED drivers (LEDD1B, Thorlabs, NJ, USA) control the light level and modulation. The photodetector (PDA36A-EC 350–1000 nm, Thorlabs, NJ, USA) has an amplifier with switchable gain that is set to 2.38 × 10^6^ V/A in this study. All components use SubMiniature version A (SMA) connectors. The data acquisition system (myDAQ, National Instruments, Berkshire, UK) has two analogue-to-digital converter (ADC) channels and two digital to analogue (DAC) channels. Only one ADC channel is used to read the output of the photodetector whilst two DAC channels are used to control the LEDs. The overall system is controlled by Labview (National Instruments, version 2015 SP1). Time-division multiple access (TDMA) is used to read both PPG signals using a single photodetector. Two 500 Hz square wave (25% duty cycle, 180° phase difference) are used to drive the two LEDs whilst the output of the photodetector is sampled at a frequency of 20 kHz.

The FBG response is measured using a SmartScan FBG interrogator (SmartScan, Smartfibres, UK) also illustrated in Figure 2. This is a high-speed interrogator with 4 channels that is capable of scanning at a frequency of 2.5 kHz for the entire wavelength range from 1528–1568 nm (i.e., over 40 nm with a resolution of 16 pm). The maximum scan frequency of the FBG interrogator with reduced wavelength range is 25 kHz.

### 2.3. Signal Processing

For the pulse oximeter, motion artefacts will affect the quality of the PPG signals collected [25]. Thus, before using the obtained PPG signals to calculate the S_p_O_2_, it is beneficial to assess the quality of these obtained PPG signals so that signals falling below a predefined quality threshold can be excluded from the analysis. There are several signal quality indices (SQIs) utilised in verifying the quality of PPG signals [22]. Two are considered here to accept or reject signals for further analysis: Perfusion Index (PI) is the most widely used [26], and skewness index (SI) which is associated with corrupted PPG signals reveals more detailed morphology of the pulse waveform [27].

PI is the ratio of the pulsatile to non-pulsatile blood volume in peripheral tissue [28]. A small PI indicates a weak pulsatile signal:

(4) $$\mathrm{PI}=\left[\frac{Y_{\max}-Y_{\min}}{\bar{x}}\right]\times 100$$

Y is the low-pass filtered PPG signal with a cut off frequency of 5 Hz. $\bar{x}$ is the absolute statistical mean of the raw PPG signal.

SI is a measure of the symmetry (or lack of it) of a probability distribution. If the pulsatile PPG signal is strong and clear, SI is positive and verse versa:

(5) $$\mathrm{SI}=\frac{1}{N}\times \overset{N}{\underset{i=N}{\sum }}{\left(x_{i}-\frac{{µ}_{x}}{\sigma }\right)}^{3}$$

N is the number of samples in the PPG signal. µ_x_ and σ are the mean and standard deviation of x_i_ respectively.

To calculate the S_p_O_2_ value, it is necessary to extract the R ratio (Equation (2)) and then use the following empirical Equation (6) [29]:

(6) $$S_{P}O_{2}=110-R\times 25$$

Although a more accurate relationship could be obtained by comparison with blood gas analysis, taking blood samples was beyond the scope of the ethical approval.

All PPG signal processing is carried out in MATLAB, version R2016a. The FBG signals are processed using the software SmartScan V3.2.0. The Bragg peaks of two FBGs in the reflection spectrum are detected using the peak detection function of the SmartScan V3.2.0.

### 2.4. FBG Calibration and Validation

In order to test the temperature response of both FBGs and the ability of FBG2 to compensate for temperature, they were both placed in an oven (ED53, Binder GmbH, Tuttlingen, Germany) and tested by heating the oven from room temperature to 49 °C. Cooling of the oven was achieved by allowing the oven, with the door open, to naturally reduce in temperature back to the laboratory ambient temperature.

**Figure 3:**
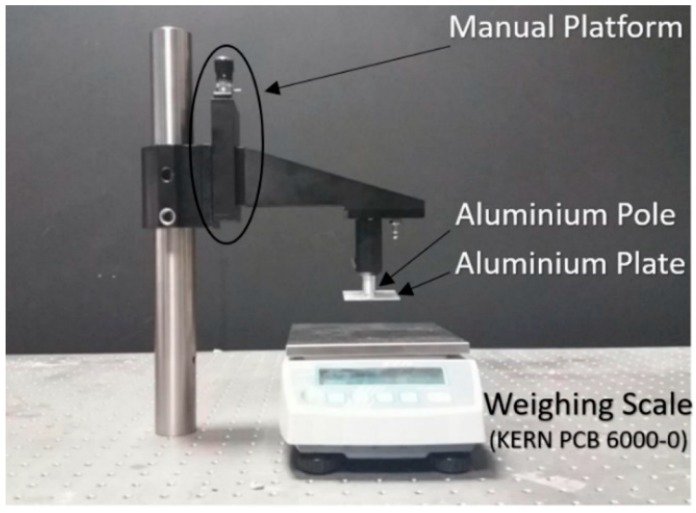
Set up for loading pressure.

The contact pressure response is demonstrated using the set up shown in Figure 3. By screwing the manual plate to lift/lower the aluminium pole and plate the pressure on the FBG patch can be adjusted. The weighing scale (PCB 6000-0, KERN & SOHN GmbH, Balingen, Germany) beneath records the force loaded on the patch and is used for calibration of the FBG sensor.

### 2.5. In Vivo Pulse Oximetry Experiments and Comparison with Commercial Devices

The designed pulse oximeter patch and a commercial transmission mode Pulse Oximeter (PO) (Radical-7, Masimo, CA, USA) simultaneously measure the S_p_O_2_ level of the index finger and the middle finger respectively. Preliminary results were obtained from a single volunteer to assess signal return and quality. A desaturation event was generated by requiring a seated volunteer to first breathe normally to obtain a baseline; then breathe out for 10–15 s; then hold breath for 20–30 s and then finally breathe normally again.

**Figure 4:**
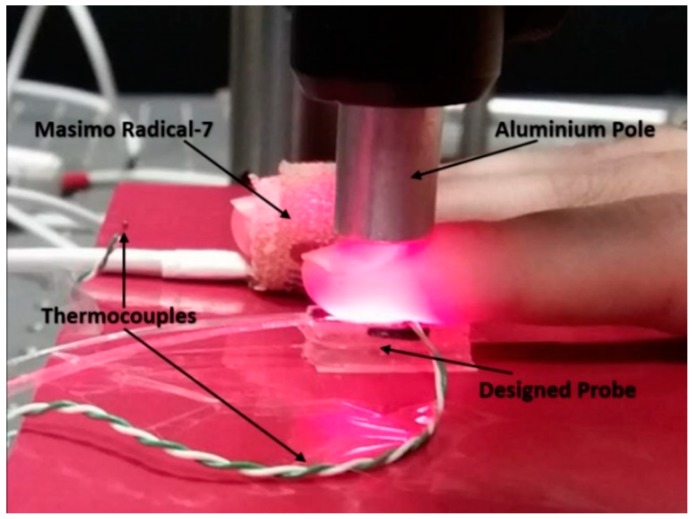
The patch beneath the index finger is the designed epoxy probe. The commercial sensor is taped on the middle finger. Two thermocouples are used to measure the environment and the index finger temperatures.

Human volunteer studies were approved by the Faculty of Engineering Ethics Committee at the University of Nottingham. Figure 4 shows the setup for experiments. The pulse oximeter and the commercial PO recorded the S_p_O_2_ value of ten volunteers’ index fingers and middle fingers. The FBG recorded the contact forces at the same time. Pressure was applied in steps (approximately 7 kPa) to the finger using the aluminium pole shown in Figure 3 and Figure 4 using the manual screw plate until the PI is too low and the pulsatile PPG component from the reflected light can no longer be observed. The pole was then gradually lifted to reduce the pressure exerted on the index finger. Each volunteer repeated the experiment three times.

Apart from the contact pressure, temperature is also an important factor. In this study, the room temperature was higher than 26 °C during experiments. This results in the temperature of the volunteers’ index fingers being higher than 30 °C (measured by PICO Technology SE000 thermocouple (Pico Technology, Saint Neots, UK)). The body temperature will affect the blood circulation and cause unwanted grating period variations. Thus, the future version of the sensor is recommended to add FBG sensors to detect the environment and subject’s temperature.

## 3. Results and Discussion

### 3.1. Preliminary S_p_O_2_ Measurement

Preliminary data showing the effectiveness and operational functionality of the system in correctly multiplexing the red and infrared channels; reducing the effects of stray light; recording PPGs and examples of the application of the signal quality indices are shown in the supporting data.

**Figure 5:**
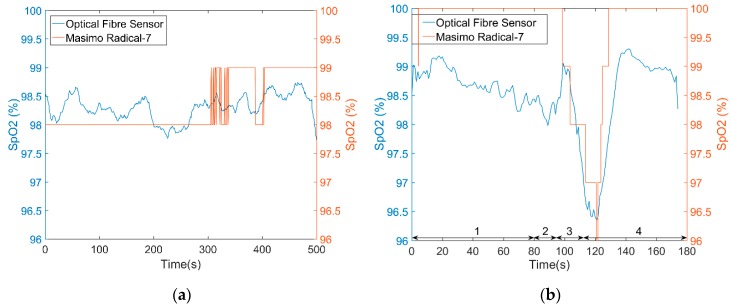
Comparison of optical fibre sensor with commercial pulse oximeter (**a**) During normal breathing both devices record stable values with the absolute error 0.443 ± 0.466%. (**b**) S_p_O_2_ test result with a desaturation event. Phase 1: Breathe normally (80 s). Phase 2: Breathe out (14 s). Phase 3: Hold breath (19 s). Phase 4: Breathe normally (66 s). Both devices identify the desaturation event with the absolute error 1.16 ± 0.423% across the trace.

Figure 5a is a S_p_O_2_ test result on one volunteer’s index finger for a period of 500 s. During the whole test, the volunteer was seated and breathed normally. A commercial pulse oximeter recorded the S_p_O_2_ value (simultaneously) of the middle finger in transmission mode and provides values to the nearest 1%. The test result of the optical fibre sensor is close to the commercial device (absolute error 0.443 ± 0.466%). Figure 5b shows a desaturation event (absolute error 1.16 ± 0.423%), although the stationary values are ~1% lower than the commercial device, the desaturation event can be easily identified.

### 3.2. FBG Pressure Sensor

Results demonstrating the effect of temperature on the FBGs and the effectiveness of the temperature reference calibration are shown in the supporting data. In this experiment, the FBG silica fibre is fixed on the optical table using tape to avoid manually straining the fibre. In future versions of the design, an additional FBG is recommended for compensating the axial strain interference.

**Figure 6:**
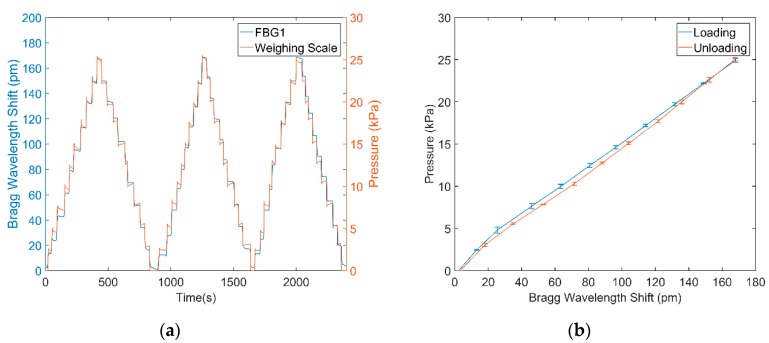
Step pressure increasing/decreasing experiment. (**a**) Increase the pressure step by step until 25 kPa. Then, the pressure decreased step by step to 0 kPa. This process was repeated three times. (**b**) Resulting FBG peak wavelength shift versus loaded pressures from Figure 6a demonstrating a linear relationship, repeatable results and low hysteresis.

Figure 6a shows the Bragg wavelength changes Δλ_FBG1_ in the FBG1 caused by pressure loading and unloading using the system shown in Figure 3. The FBG response follows that of the applied pressure. Using the data in Figure 6a the relationship between applied pressure and wavelength can be obtained by plotting a calibration curve as shown in Figure 6b. This indicates that the FBG patch is both reliable and repeatable for pressure monitoring with low hysteresis. Based on Figure 6b, the empirical equation of pressure calculation can be deduced:

(7) $$\mathrm{Pressure}\left(\mathrm{kPa}\right)=0.1466\times {\Delta \lambda }_{FBG1}+0.7334$$

### 3.3. In Vivo Pulse Oximetry and Pressure Monitoring

Typical results for a single volunteer are presented to demonstrate the fibres pulse oximetry response to applied pressure, followed by a summary of the errors due to applied pressure for all volunteers. The figures of all 30 experimental results are illustrated in the supporting file (Figures S6–S35). Figure 7a shows the infrared PPG signal (red curve) and the contact pressure (blue curve). As contact pressure increases the AC component of the PPG signal decreases (i.e., a thinner red line) and the DC light level increases. This is to be expected as the blood flow is occluded and the tissue blanches.

**Figure 7:**
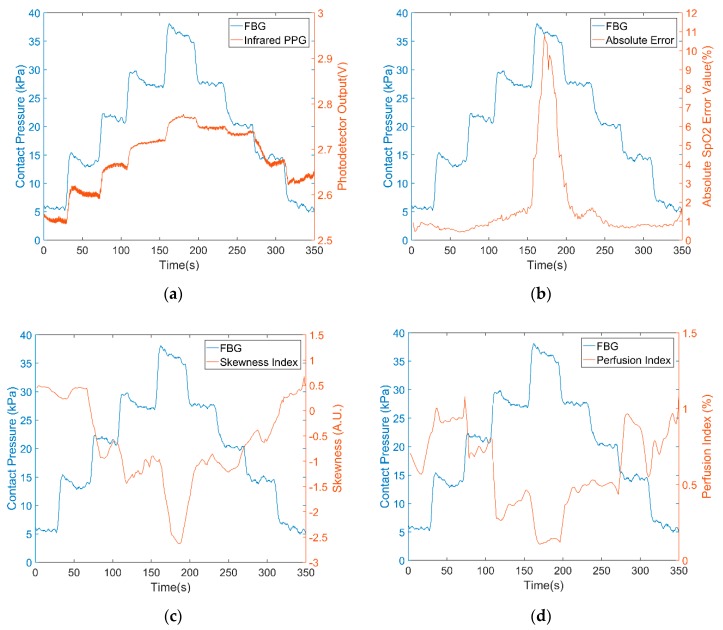
(**a**) Infrared PPG signals (red) under different contact pressures (blue). (**b**) Absolute S_P_O_2_ error (red) compared to an unloaded commercial device (**c**) skewness index of infrared PPG (red) (**d**) Perfusion index of infrared PPG (red).

Figure 7b shows the absolute S_p_O_2_ error between the optical fibre sensor and the commercial device. The initial pressure generated by the weight of the index finger was around 4 to 6 kPa. For pressures higher than 15 kPa, the reliability and accuracy of the sensor for S_p_O_2_ determination are degraded. Figure 7c,d reinforce this degradation by illustrating the PI and SI of the infrared PPG signals falling. When the contact pressure was increased higher than 15 kPa, both PI and SI sharply decrease but recover when the pressure returns below 15 kPa.

**Figure 8:**
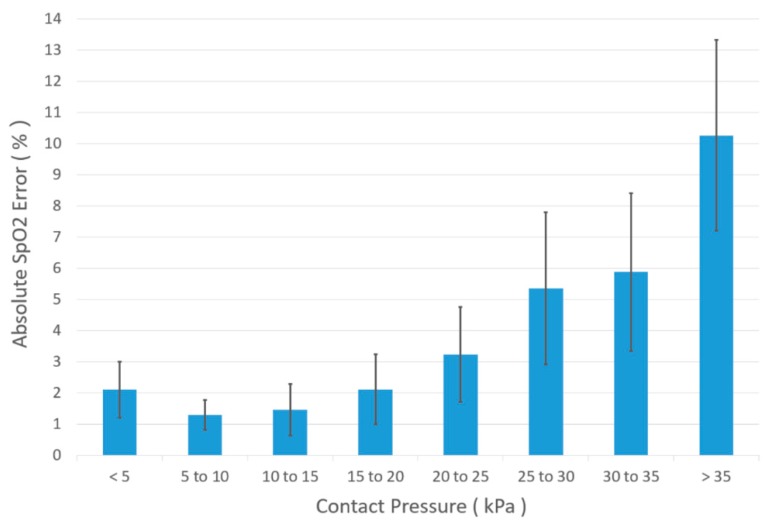
Effect of contact pressure on S_p_O_2_ error between optical fibre sensor and a commercial pulse oximeter. When the contact pressure is higher than 25 kPa, the S_p_O_2_ error is >10%. When the contact pressure is lower than 15 kPa, the S_p_O_2_ error is lower than 2%. The S_p_O_2_ error reached the lowest value while the contact pressure is in the range from 5 to 15 kPa.

For each of the ten volunteers, this experiment was repeated three times to provide 30 datasets, all of which were similar to those shown in Figure 7a–d. By averaging three measurements from the ten volunteers, a bar chart was established (see Figure 8) which shows the contact pressure effects on S_p_O_2_ measurement. Here, we can see that the pressure range from 5 to 15 kPa is the most suitable for S_p_O_2_ monitoring. This pressure range is close to the largest PPG amplitude obtained pressure range (8 to 12 kPa) for a reflectance sensor attached to the forehead region above the eye [30,31,32]. Thus, in future studies, it is suggested to apply the designed sensor to measure the forehead S_p_O_2_ value.

Since the different modes of two pulse oximeters applied (reflectance/transmission mode) contributes to the S_p_O_2_ error as well, in future studies, the algorithm chosen for calculating S_p_O_2_ should be modified to reduce the error between the designed sensor and the commercial pulse oximeter.

## 4. Conclusions

A novel integrated optical fibre sensor probe combining a reflectance pulse oximeter with a fibre Bragg grating contact pressure sensor has been demonstrated. To maximise light delivery and collection to and from the tissue, each plastic optical fibre used in the pulse oximeter is cleaved at 45° at its distal end. All optical fibres are housed in a biocompatible epoxy patch which serves two purposes: (i) to reduce motion artefacts in the photoplethysmogram; and (ii) to transduce transverse loading into an axial strain in the FBG. Test results show that using a combination of pressure measuring FBG with a reference FBG, reliable results are possible with low hysteresis.

In vivo pulse oximetry experiments on 10 healthy volunteers demonstrated the effect of contact pressure on S_p_O_2_ monitoring. Based on the S_p_O_2_ error between a commercial pulse oximeter and the designed sensor along with two signal quality indices, the optimum pressure range for S_p_O_2_ detection was found to lie between 5 kPa and 15 kPa.

The combined probe has the potential to improve the reliability of reflectance oximeters. In particular in wearable technology the probe should find use in optimising the fitting of garments incorporating this technology. Often garments worn are tightly fitting which can limit user adherence and applying the optimum pressure without being too tight could be beneficial. The probe also has the potential to be used in loosely fitting garments where measurements are only recorded when the appropriate pressure is applied.

## Supplementary Materials

The following are available online at http://www.mdpi.com/1424-8220/18/11/3632/s1: Figure S1: TDMA system time slot; Figure S2: The immunity of the POF patch to stray light interference; Figure S3: Reflected PPG signals obtained by the POF patch; Figure S4: SQIs of high, medium and low quality PPG signals; Figure S5: Temperature performance of FBG 1 and 2; Figures S6–S35: 30 groups of experiments data recorded.
