# Supplementary material for: Optical Fibre-Based Pulse Oximetry Sensor with Contact Force Detection
Source: Sensors (Basel). 2018 Oct 26;18(11):3632. doi: 10.3390/s18113632 (PMC6263952; doi:10.3390/s18113632)
Supplement: Supplementary file 1 [file sensors-18-03632-s001.pdf]

SUPPORTING DATA

Optical Fibre Based Pulse Oximetry Sensor with Contact Force Detection

1) *Time division multiplexing*

Figure S1a shows outputs of two DAC channels of the DAQ system. These two square wave signals switched on/off the red and the infrared LEDs. Figure S1b shows the output of two LEDs detected by the photodetector. From the figure S2.b, it is clear that two LED outputs do not overlap. According to the result, the system is capable of using one photodetector to detect dual PPG signals.

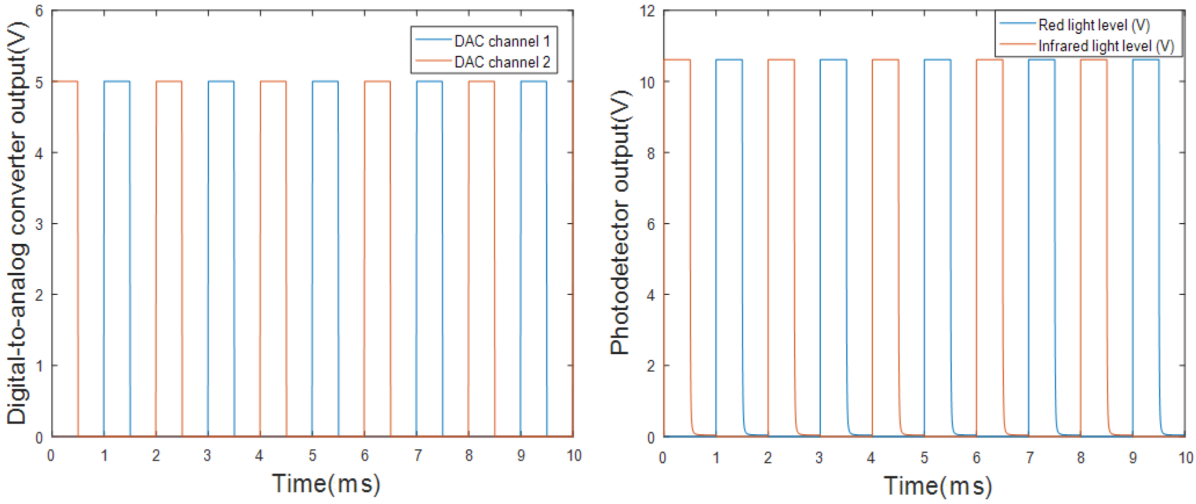

(a) (b)

S 1. (a) TDMA system time slot. These two square waves have 180° phase difference. The red square wave controls the red LED and the blue square wave controls the infrared LED. (b) Optical signal detected by the photodetector. The photodetector detects light emitted from two LEDs directly which are transferred by POFs. The blue one is the output of the red LED controlled by DAC channel 1 and the red one is the output of the infrared LED controlled by DAC channel 2.

2) *Preliminary PPG measurements*

Figure S2 shows the immunity of the PPG sensor to stray light. Using the configuration shown in Figure 2, both LEDs were initially switched off and the only light present was room light. After 30s, both LEDs were switched on which resulted in a 0.006 mV increase in light intensity level caused by light directly passing from illumination to detector fibre. Putting the index finger on the POF patch results in a significant increase in detected light. Figure S3 shows typical reflected PPG signals obtained by the sensor.

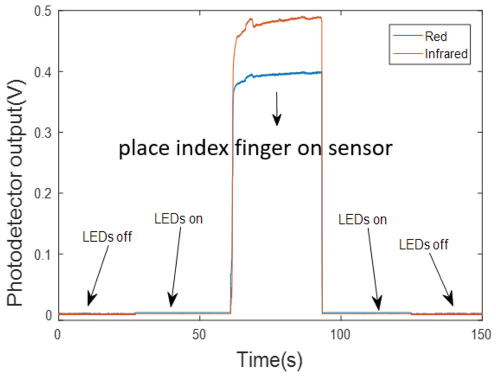

S 2. The immunity of the patch to stray light. The black bar and the black material absorb the most of the stray light. Compared to the light level of PPG signals, the stray light effect is negligible.

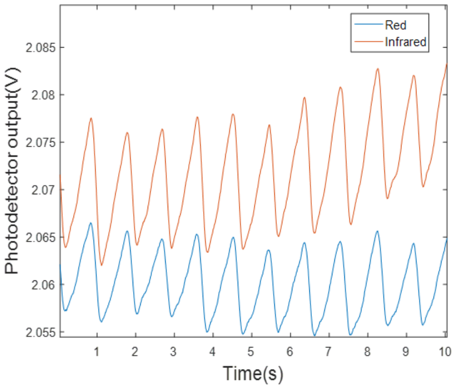

S 3. Reflected PPG signals obtained by the sensor. After data processing, two different wavelength PPG signals are separated.

As an example of the application of the two signal quality indices, three signal types are shown in figure S4 corresponding qualitatively to high (top row), medium (middle row) and low (bottom row) quality signals. If the pulsatile component of the PPG signal is too weak, the PI value is very small. If the pulsatile component of the PPG signals is not clear enough to identify systolic and diastolic peaks, the SI value is negative.

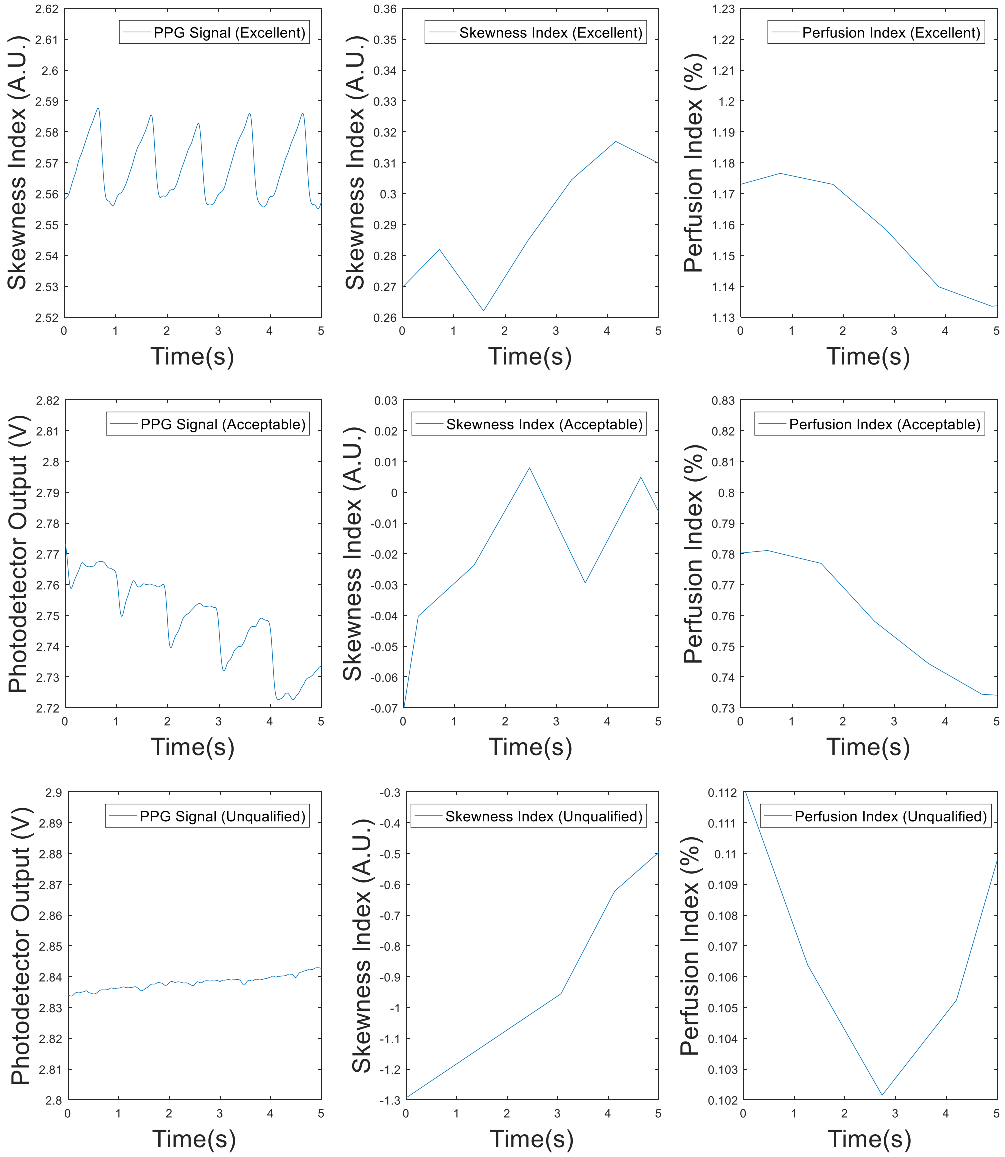

S 4. SQIs of high (top row), medium (middle row) and low (bottom row) quality PPG signals. For high quality signals, the PI value is high and the SI value is positive. When the PPG signal quality gets worse, both PI and SI values decrease.

Due to empirical experience, the PI value of good quality PPG signals is usually larger than 0.4%. Therefore, in this report, the threshold of PI is set at 0.4% and the threshold of SI is zero.

3) FBG Temperature Compensation

Figure S5 shows the temperature response of FBG1 (pressure sensor) and FBG2 (reference). The peak wavelength shift curves of both FBGs follows the same trend. Therefore, the Bragg wavelength shift of the FBG2 can be directly subtracted from FBG1 to compensate temperature. The empirical equation 6 describes a simple temperature compensation calculation.

$$\lambda_{\Delta P\text{-}FBG1} = \lambda_{\Delta FBG1} - 2.7577 \times \lambda_{\Delta FBG2} + 0.0147 \quad (6)$$

$\lambda_{\Delta P\text{-}FBG1}$ is the Bragg wavelength shift in the FBG sensor 1 with temperature compensation. $\lambda_{\Delta FBG1}$ and $\lambda_{\Delta FBG2}$ are Bragg wavelength shifts read from the interrogator.

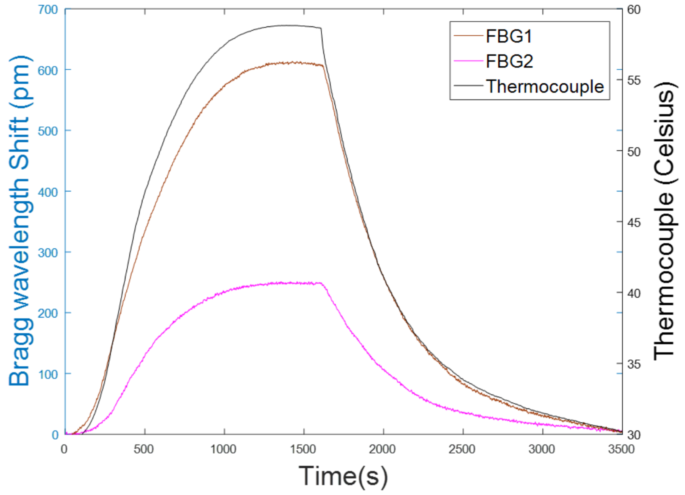

S 5. Temperature performance of FBG 1&2. Pink and brown curves are the Bragg (peak) wavelength shift of FBG 1&2 ranging from 30°C to 60°C. The black line presents the output of the themocouple. The peak wavelength of both FBGs shift in accordance with the temperature changes.

4) Experimental results from 10 volunteers.

In this study, the designed pulse oximetry sensor was applied to measure the S_PO_2 value of 10 volunteers under different contact pressure levels. For each volunteer, the experiment is required to repeat the whole pressure changing process for three times. Therefore, there are 30 groups of data recorded in total which are shown from S6 to S35. Every figure in the range from S6 to S35 contains 4 diagrams which show the infrared PPG signal (a), the absolute S_PO_2 error (b), the skewness index (c) and the perfusion index (d) under different pressure levels.

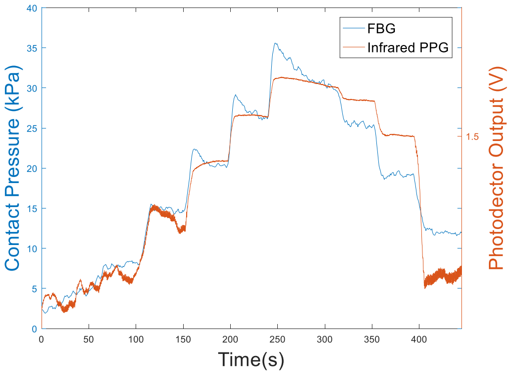

(a)

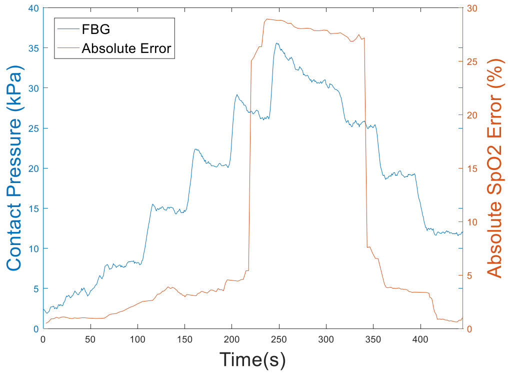

(b)

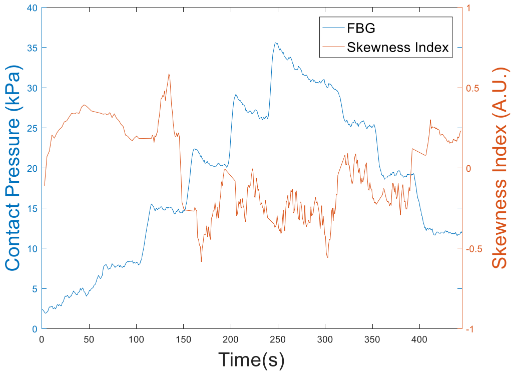

(c)

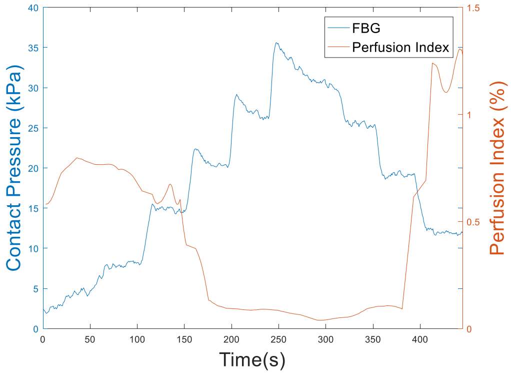

(d)

S 6. Volunteer 1-1. (a) FBG VS. Infrared PPG signals. (b) FBG VS. Absolute S_PO_2 error. (c) FBG VS. Skewness index. (d) FBG VS. Perfusion index.

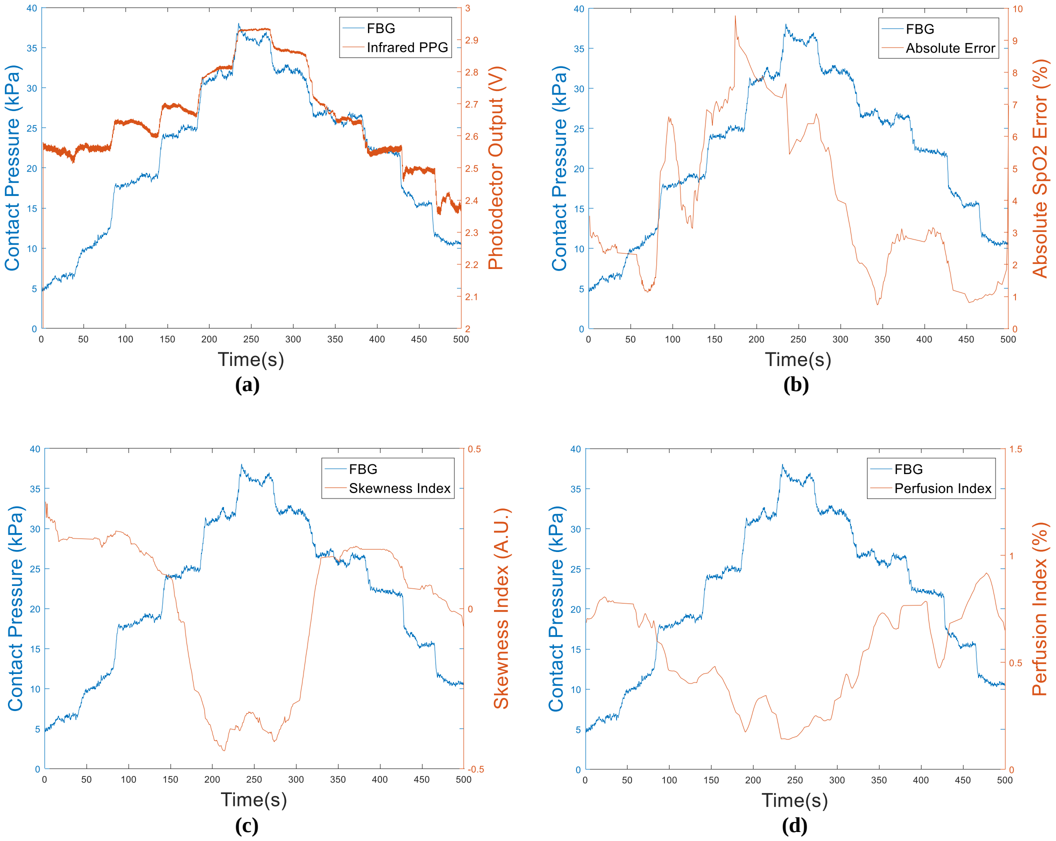

S 7. Volunteer 1-2. (a) FBG VS. Infrared PPG signals. (b) FBG VS. Absolute S_PO_2 error. (c) FBG VS. Skewness index. (d) FBG VS. Perfusion index.

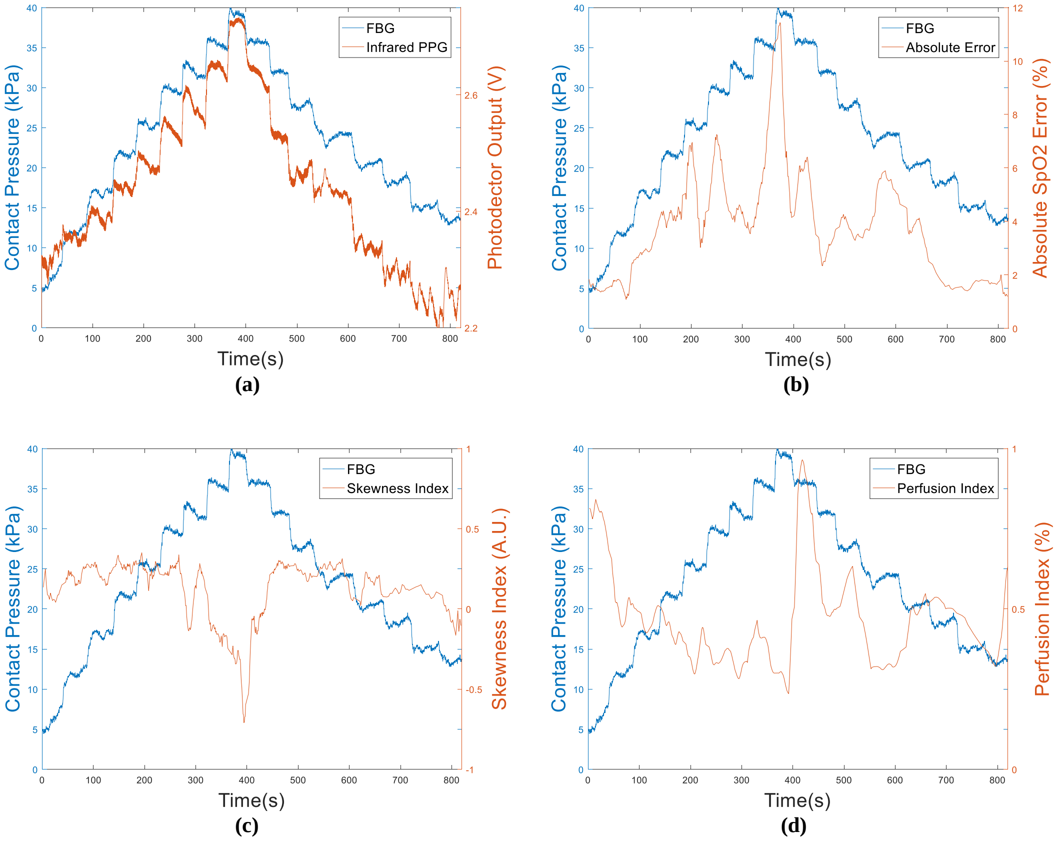

S 8. Volunteer 1-3. (a) FBG VS. Infrared PPG signals. (b) FBG VS. Absolute S_PO_2 error. (c) FBG VS. Skewness index. (d) FBG VS. Perfusion index.

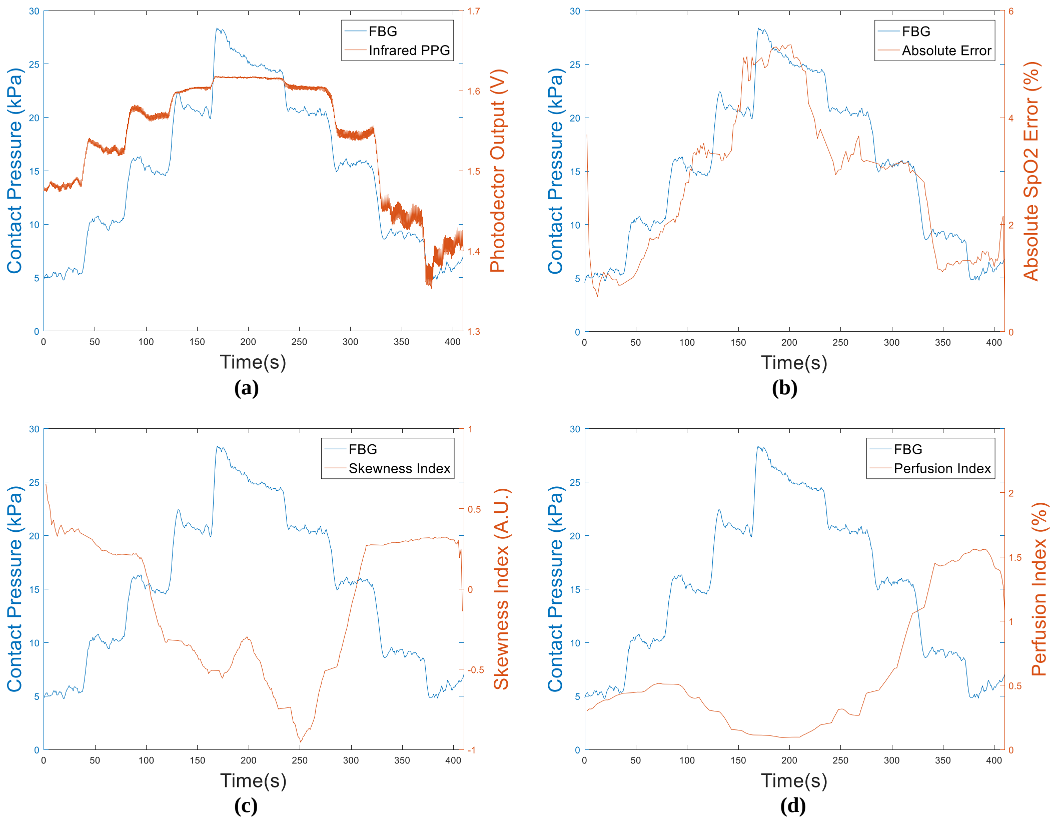

S 9. Volunteer 2-1. (a) FBG VS. Infrared PPG signals. (b) FBG VS. Absolute S_PO_2 error. (c) FBG VS. Skewness index. (d) FBG VS. Perfusion index.

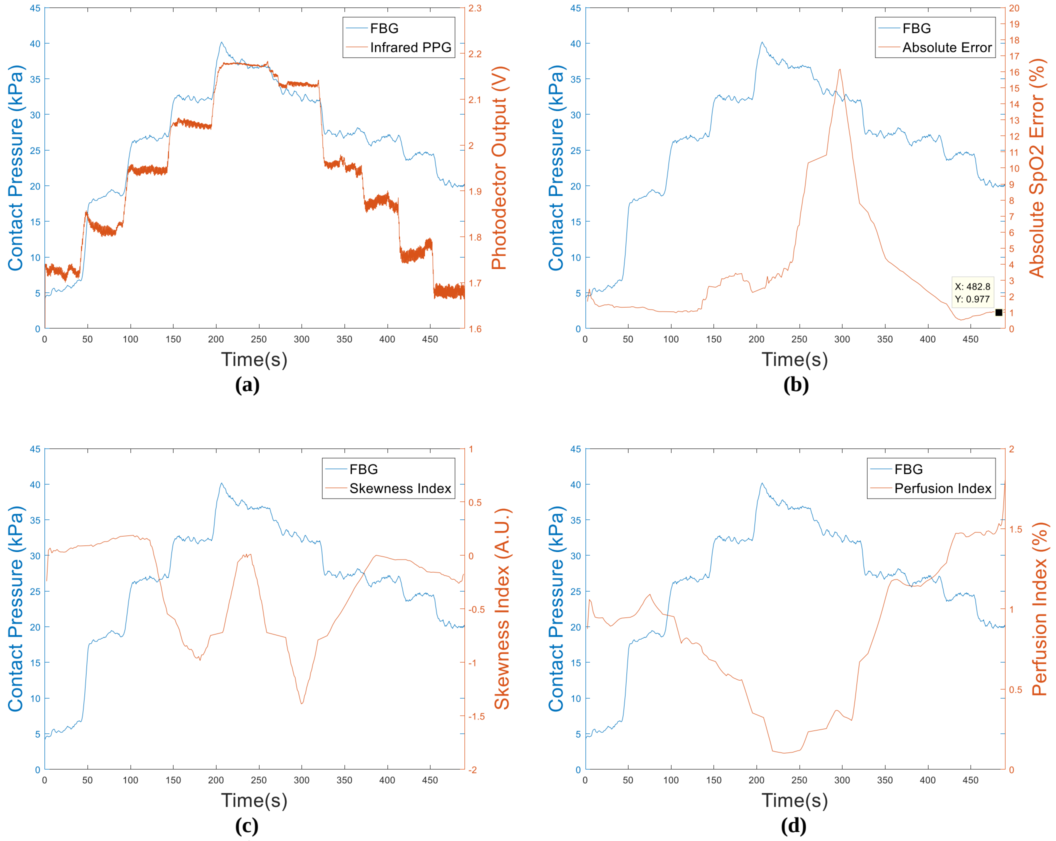

S 10. Volunteer 2-2. (a) FBG VS. Infrared PPG signals. (b) FBG VS. Absolute S_PO_2 error. (c) FBG VS. Skewness index. (d) FBG VS. Perfusion index.

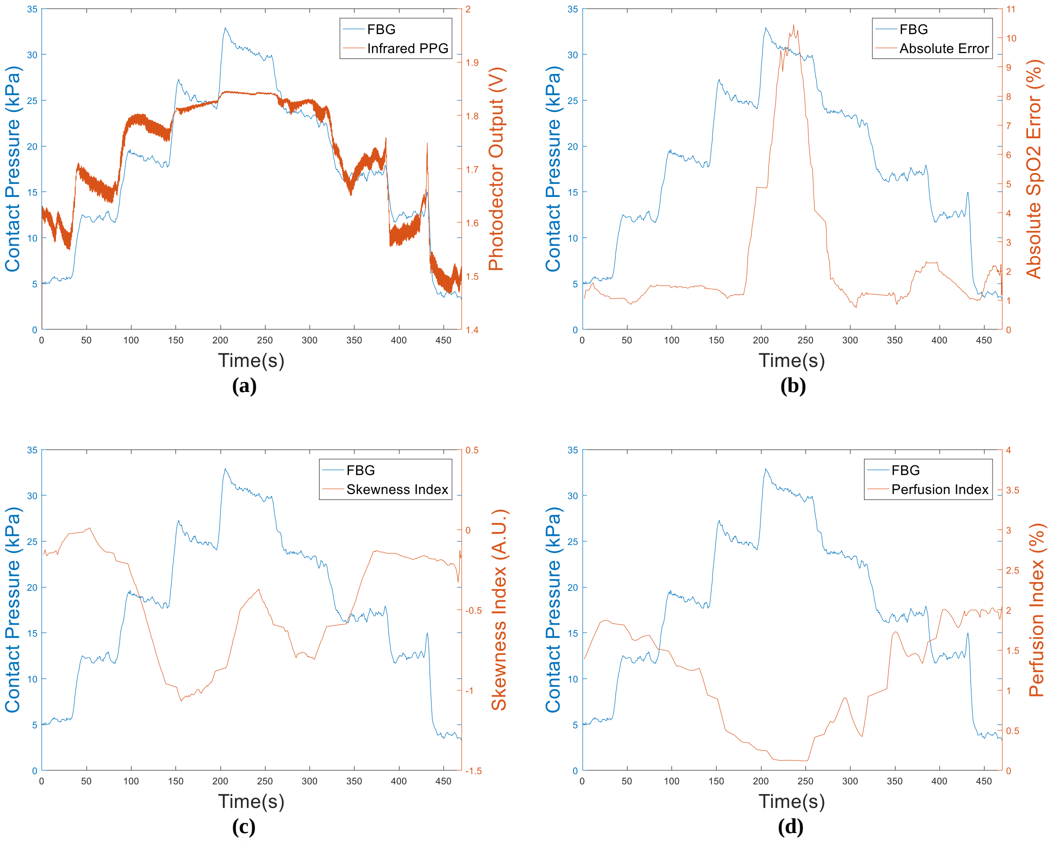

S 11. Volunteer 2-3. (a) FBG VS. Infrared PPG signals. (b) FBG VS. Absolute S_PO_2 error. (c) FBG VS. Skewness index. (d) FBG VS. Perfusion index.

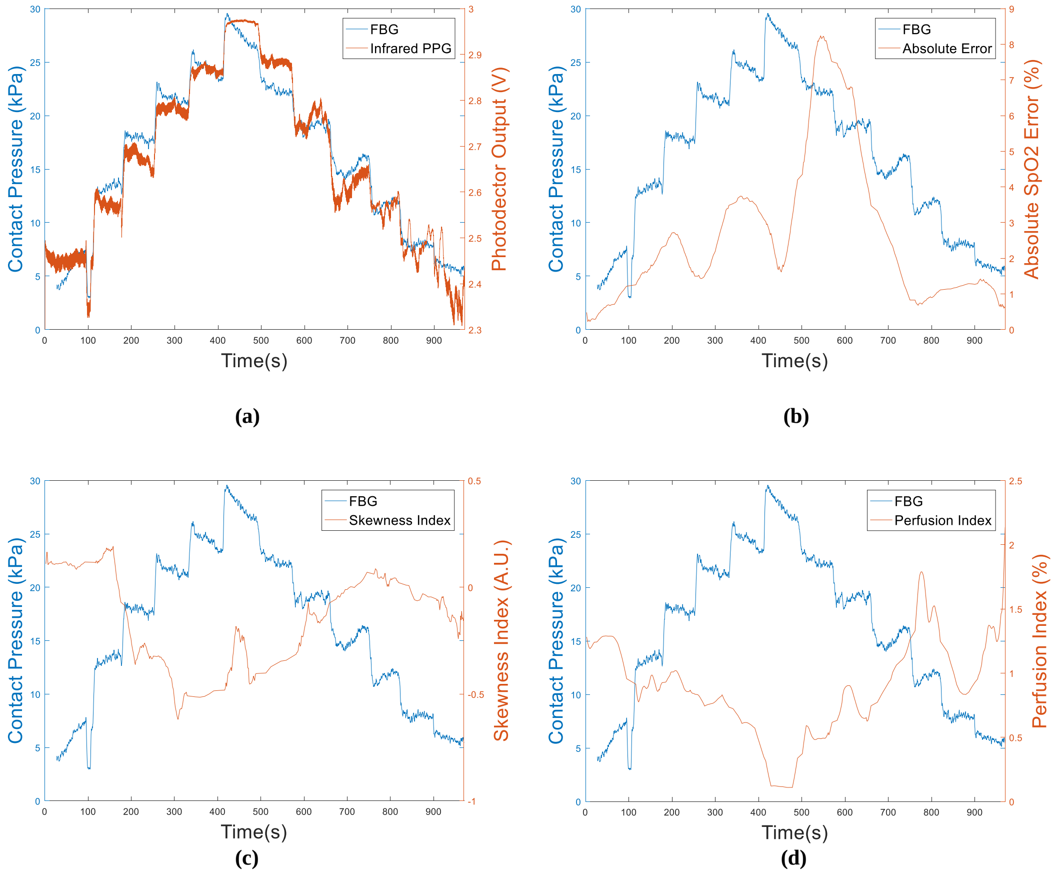

S 12. Volunteer 3-1. (a) FBG VS. Infrared PPG signals. (b) FBG VS. Absolute S_PO_2 error. (c) FBG VS. Skewness index. (d) FBG VS. Perfusion index.

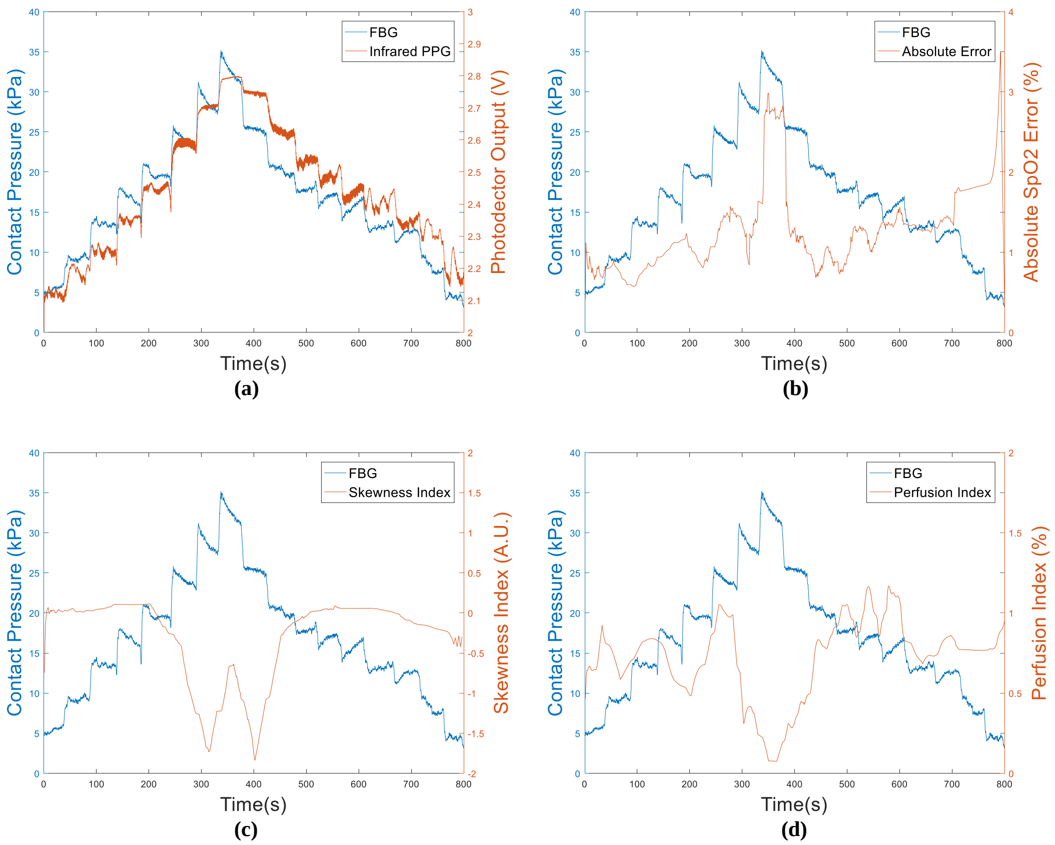

S 13. Volunteer 3-2. (a) FBG VS. Infrared PPG signals. (b) FBG VS. Absolute S_PO_2 error. (c) FBG VS. Skewness index. (d) FBG VS. Perfusion index.

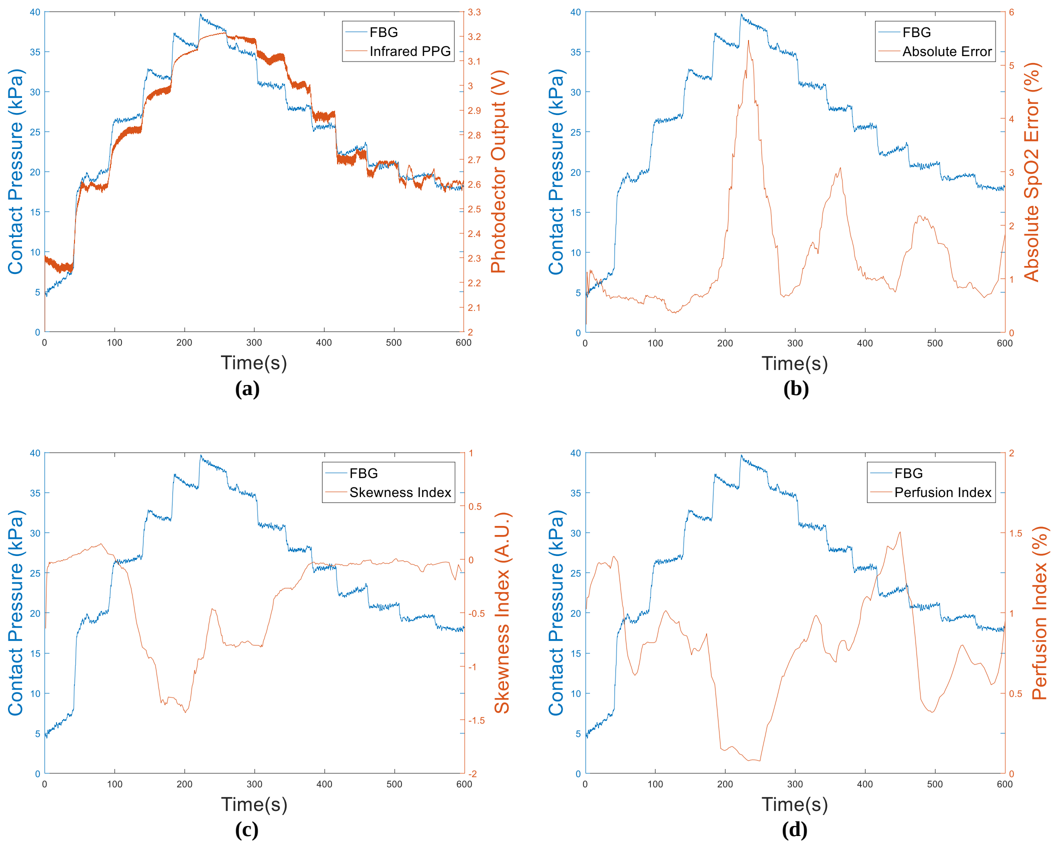

S 14. Volunteer 3-3. (a) FBG VS. Infrared PPG signals. (b) FBG VS. Absolute S_PO_2 error. (c) FBG VS. Skewness index. (d) FBG VS. Perfusion index.

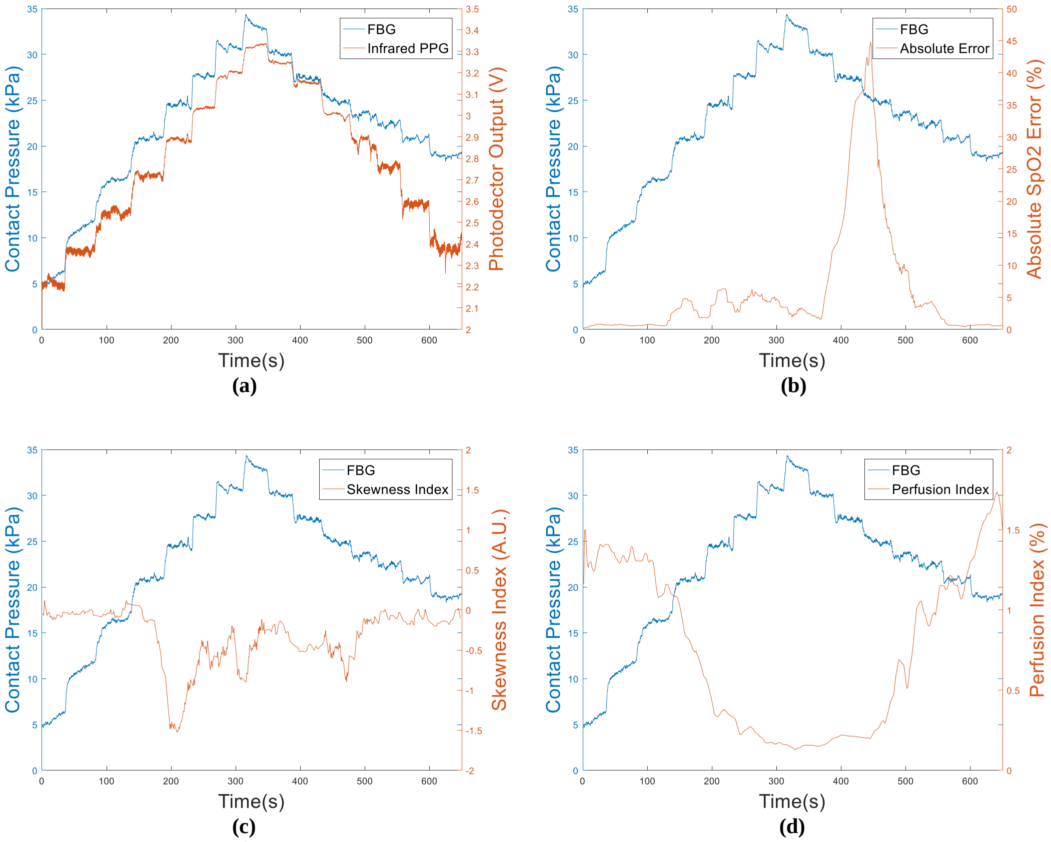

S 15. Volunteer 4-1. (a) FBG VS. Infrared PPG signals. (b) FBG VS. Absolute S_PO_2 error. (c) FBG VS. Skewness index. (d) FBG VS. Perfusion index.

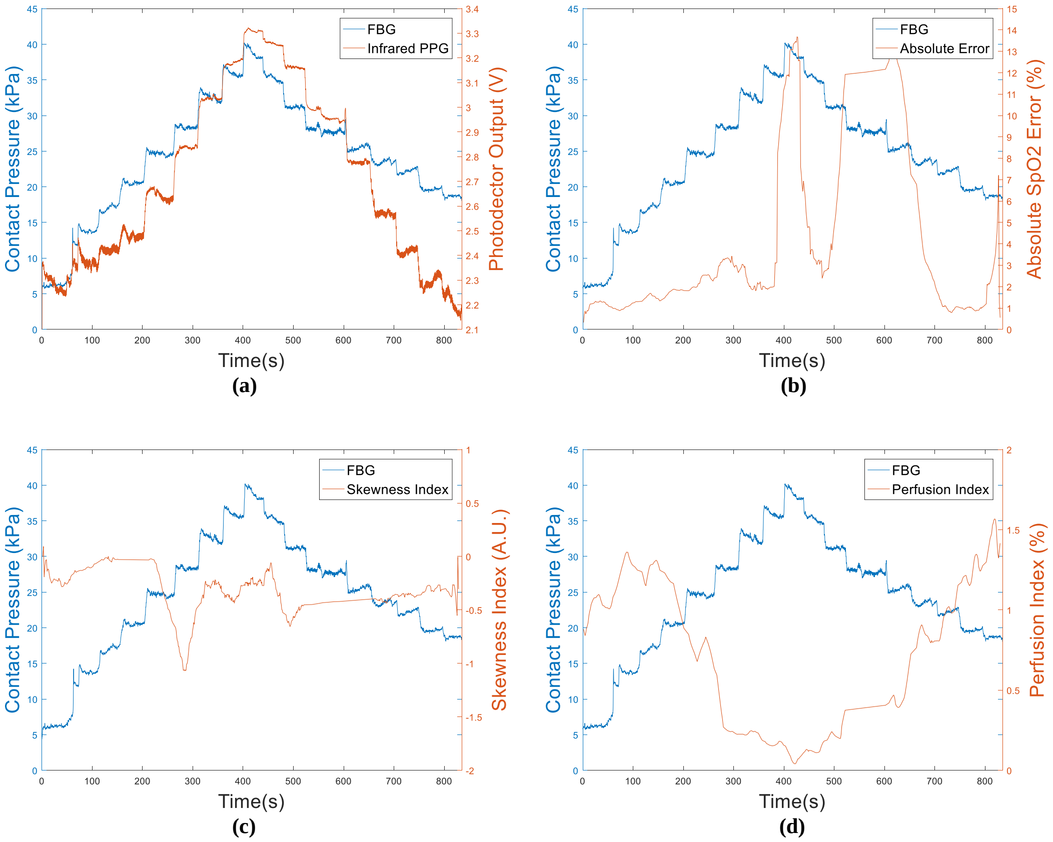

S 16. Volunteer 4-2. (a) FBG VS. Infrared PPG signals. (b) FBG VS. Absolute S_PO_2 error. (c) FBG VS. Skewness index. (d) FBG VS. Perfusion index.

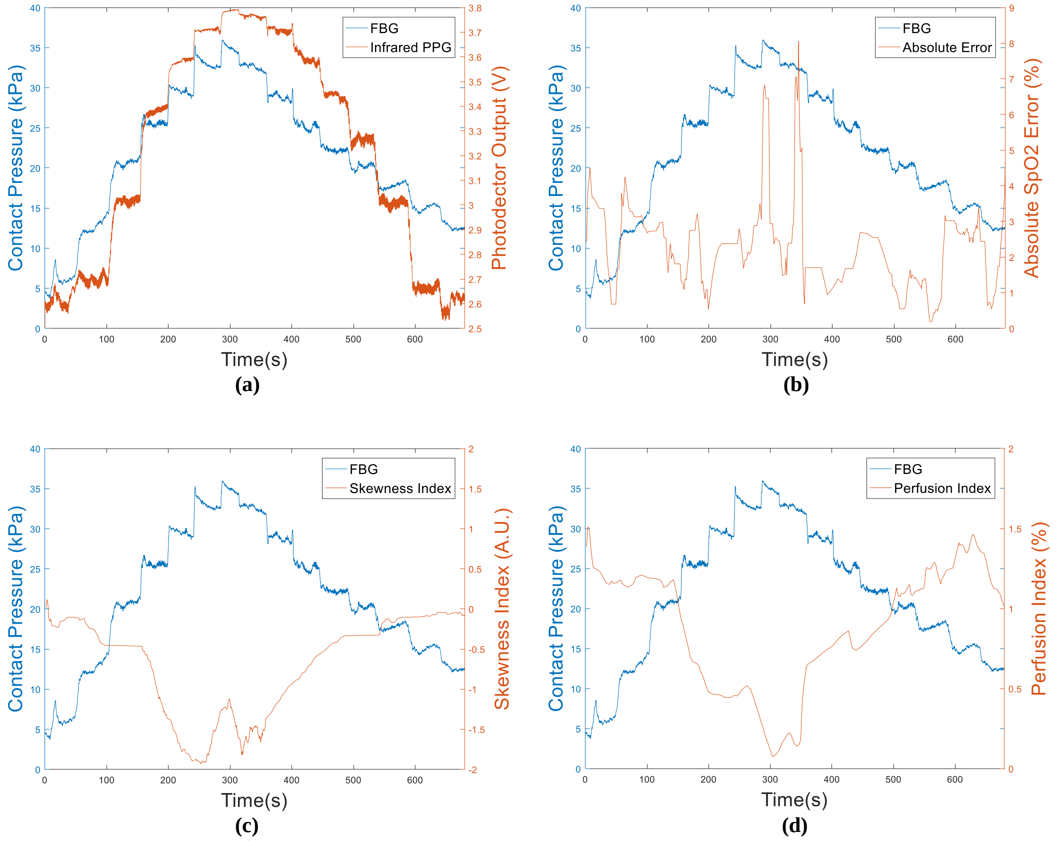

S 17. Volunteer 4-3. (a) FBG VS. Infrared PPG signals. (b) FBG VS. Absolute S_PO_2 error. (c) FBG VS. Skewness index. (d) FBG VS. Perfusion index.

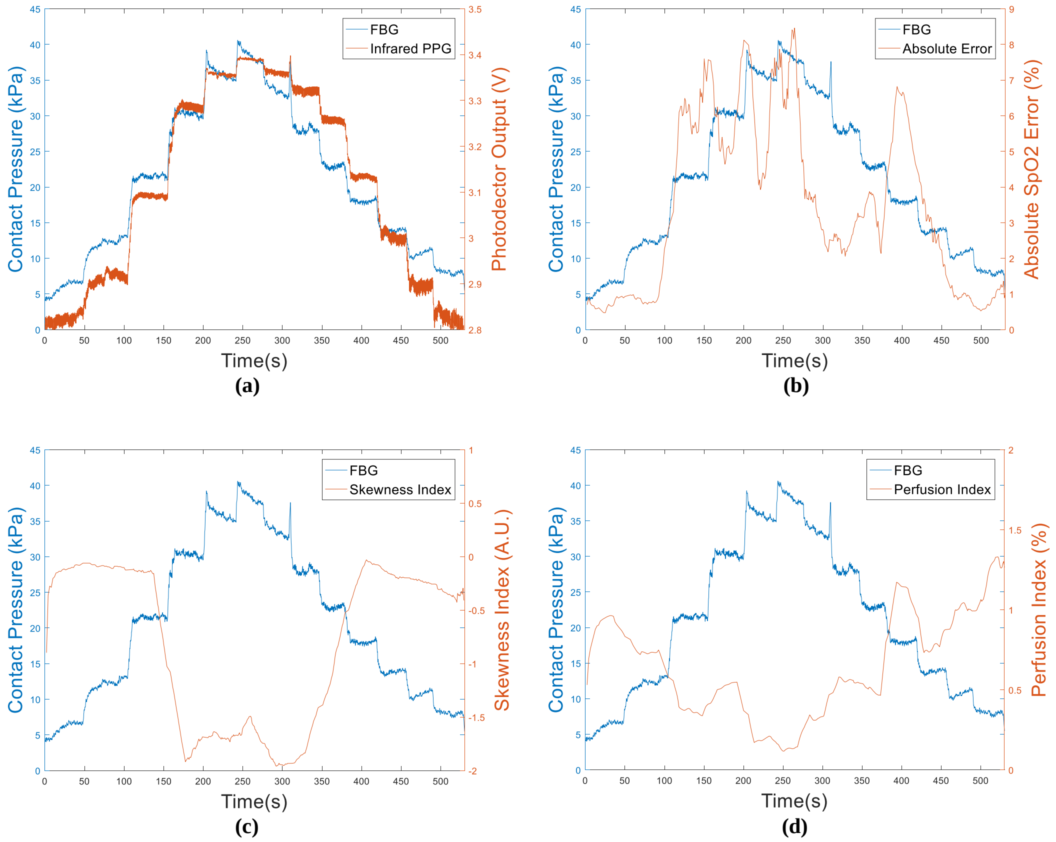

S 18. Volunteer 5-1. (a) FBG VS. Infrared PPG signals. (b) FBG VS. Absolute S_PO_2 error. (c) FBG VS. Skewness index. (d) FBG VS. Perfusion index.

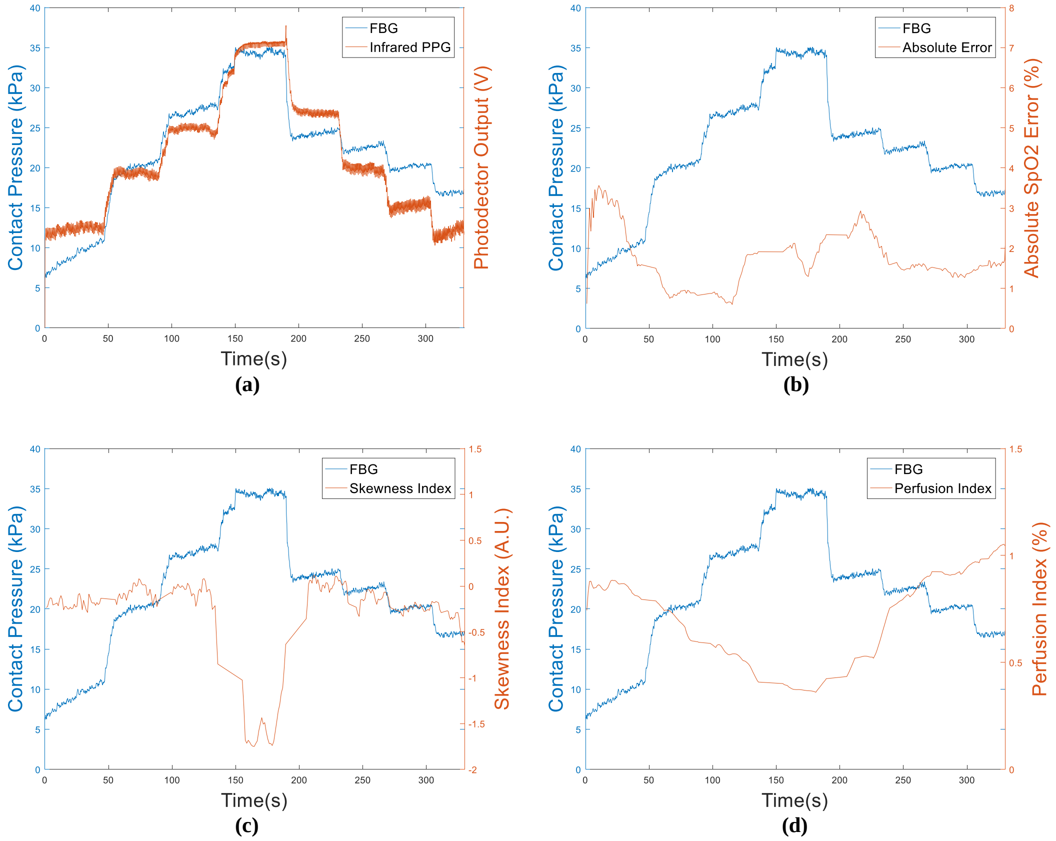

S 19. Volunteer 5-2. The connection between the opto-electronic system and which generated the gap existed in the experiment results. (a) FBG VS. Infrared PPG signals. (b) FBG VS. Absolute S_PO_2 error. (c) FBG VS. Skewness index. (d) FBG VS. Perfusion index.

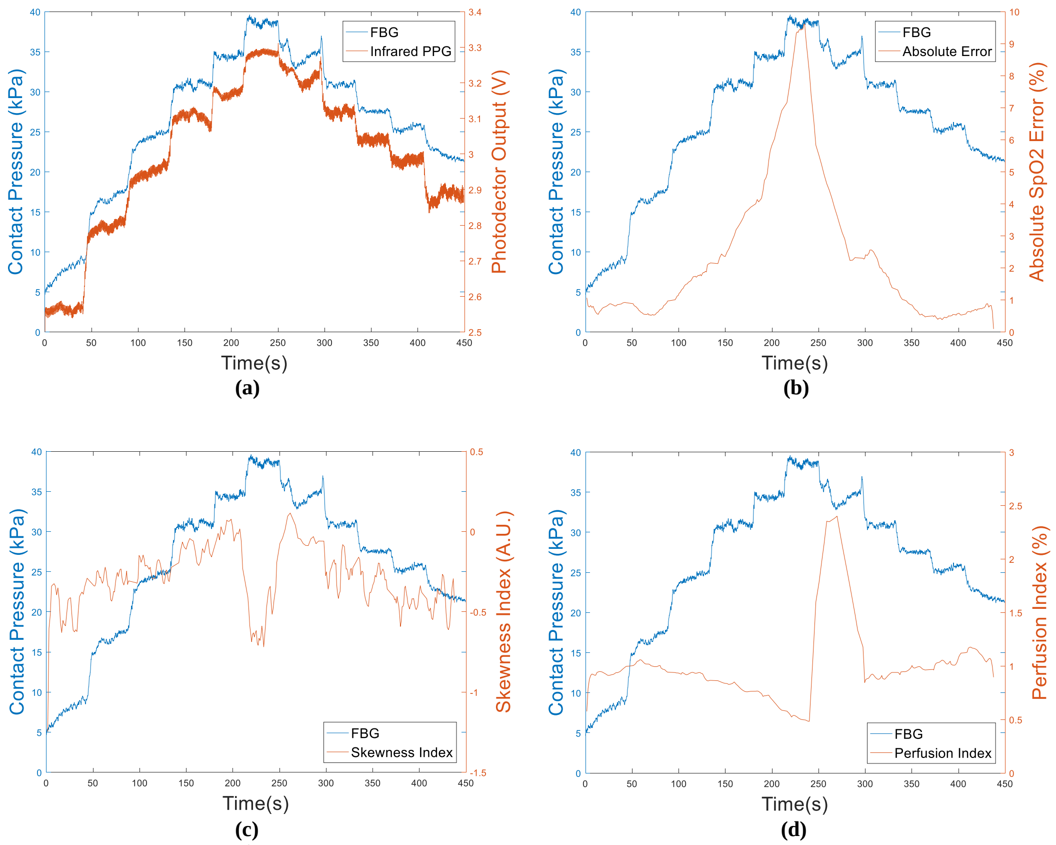

S 20. Volunteer 5-3. (a) FBG VS. Infrared PPG signals. (b) FBG VS. Absolute S_PO_2 error. (c) FBG VS. Skewness index. (d) FBG VS. Perfusion index.

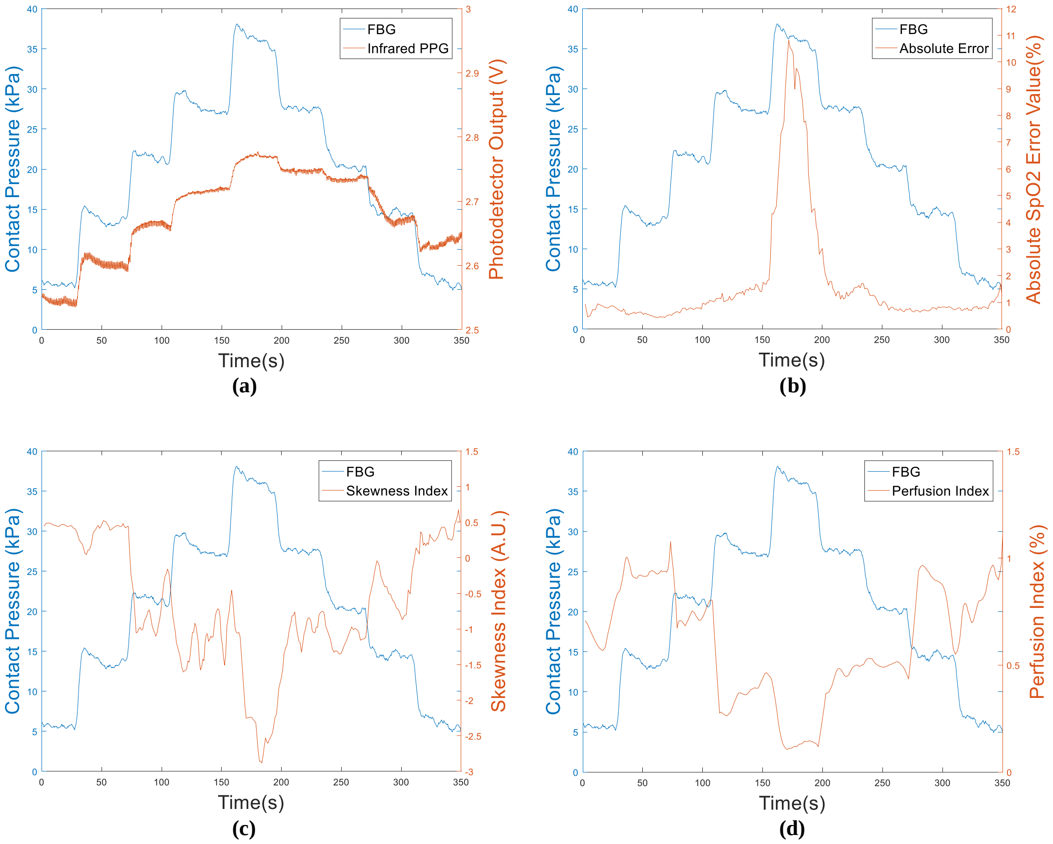

S 21. Volunteer 6-1. (a) FBG VS. Infrared PPG signals. (b) FBG VS. Absolute S_PO_2 error. (c) FBG VS. Skewness index. (d) FBG VS. Perfusion index.

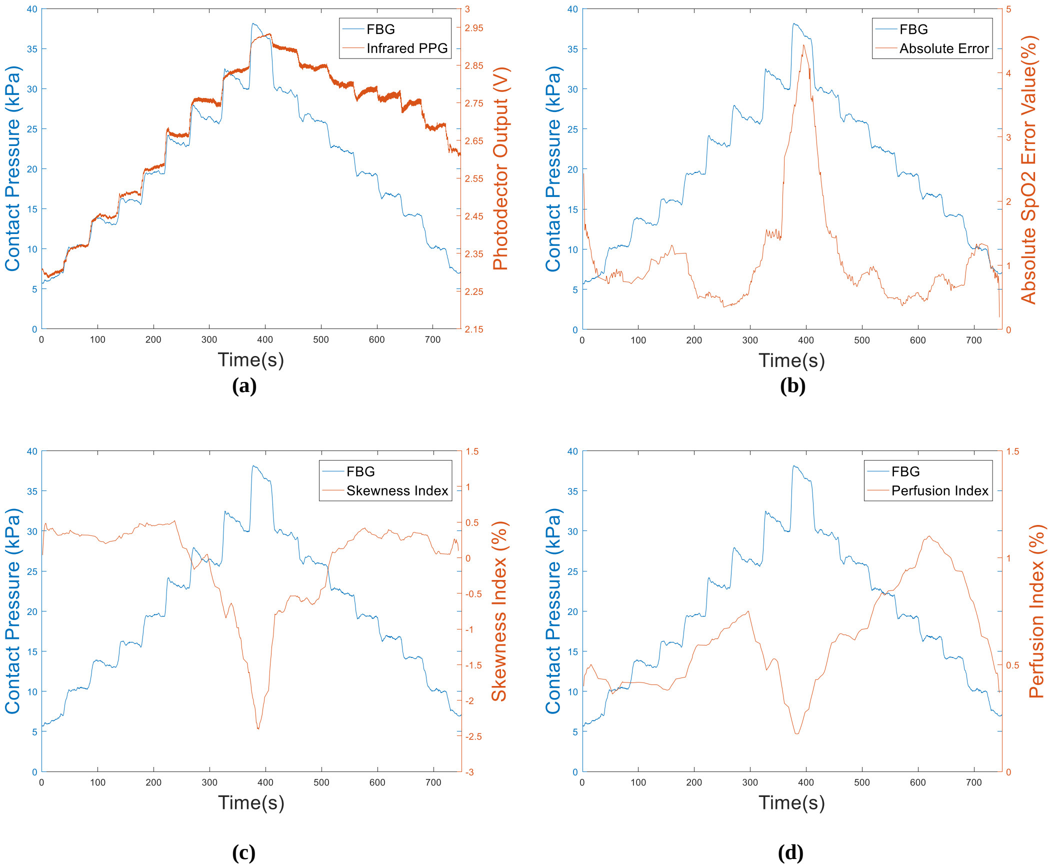

S 22. Volunteer 6-2. (a) FBG VS. Infrared PPG signals. (b) FBG VS. Absolute S_PO_2 error. (c) FBG VS. Skewness index. (d) FBG VS. Perfusion index.

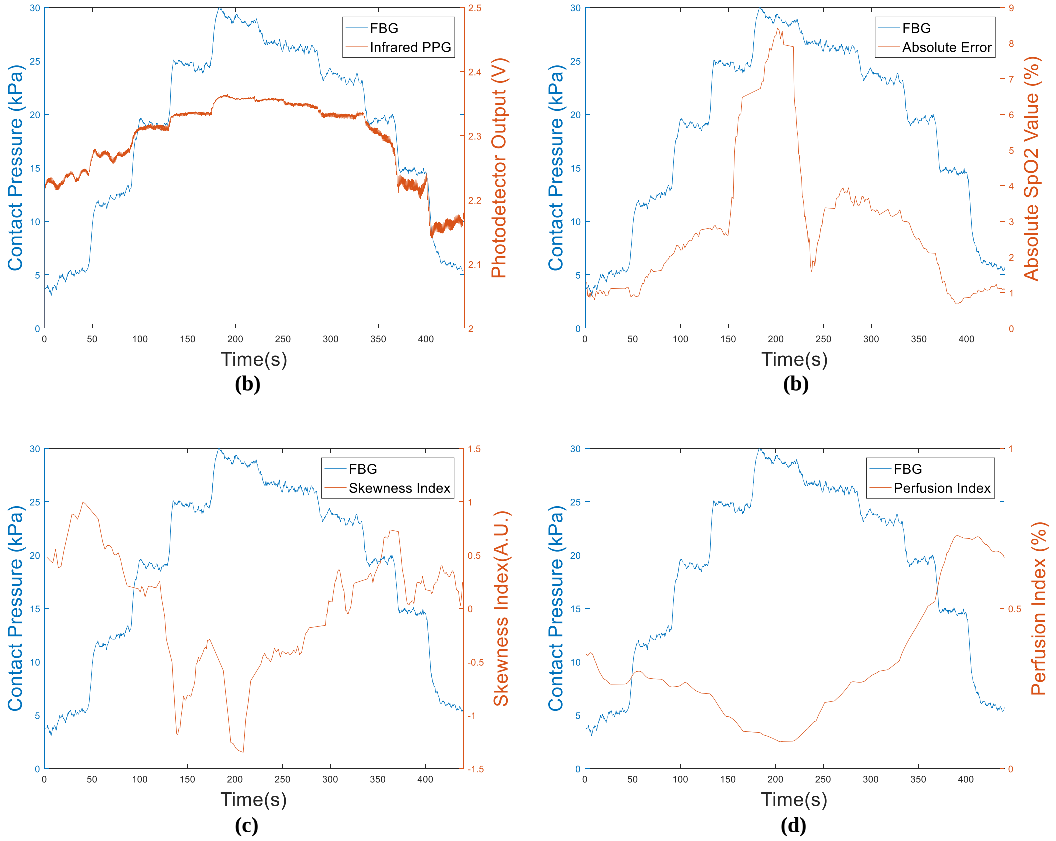

S 23. Volunteer 6-3. (a) FBG VS. Infrared PPG signals. (b) FBG VS. Absolute S_PO_2 error. (c) FBG VS. Skewness index. (d) FBG VS. Perfusion index.

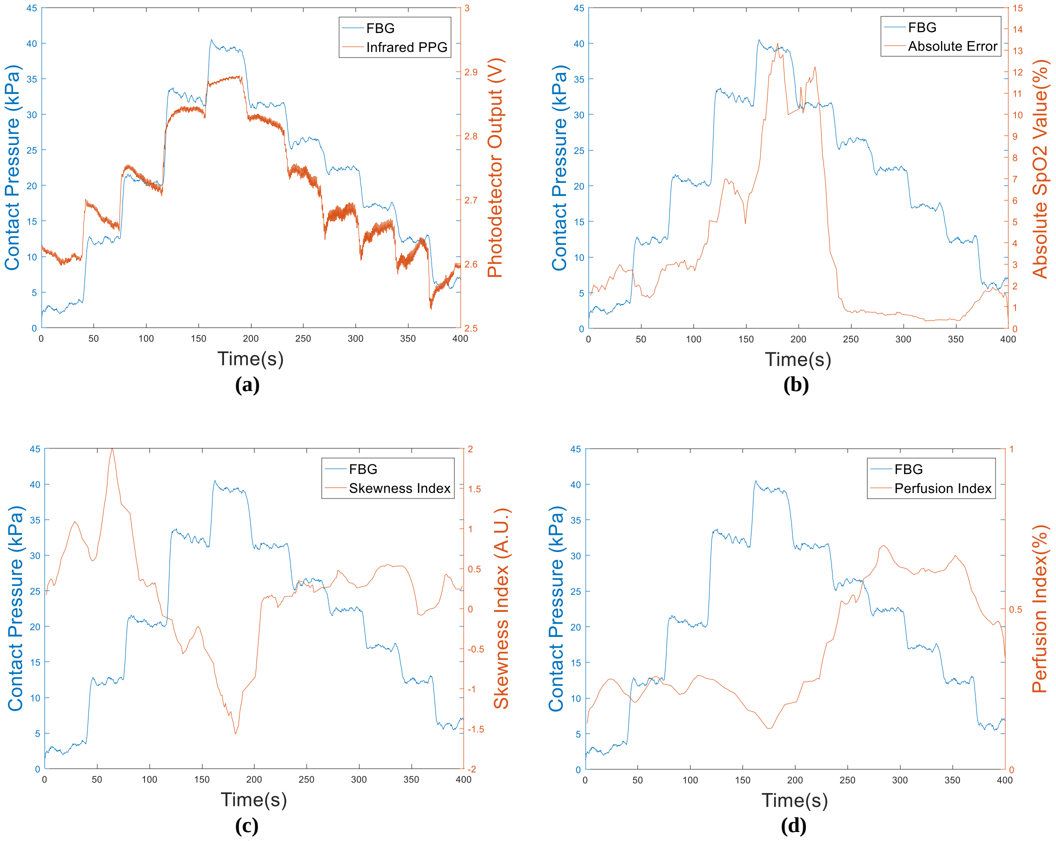

S 24. Volunteer 7-1. (a) FBG VS. Infrared PPG signals. (b) FBG VS. Absolute S_PO_2 error. (c) FBG VS. Skewness index. (d) FBG VS. Perfusion index.

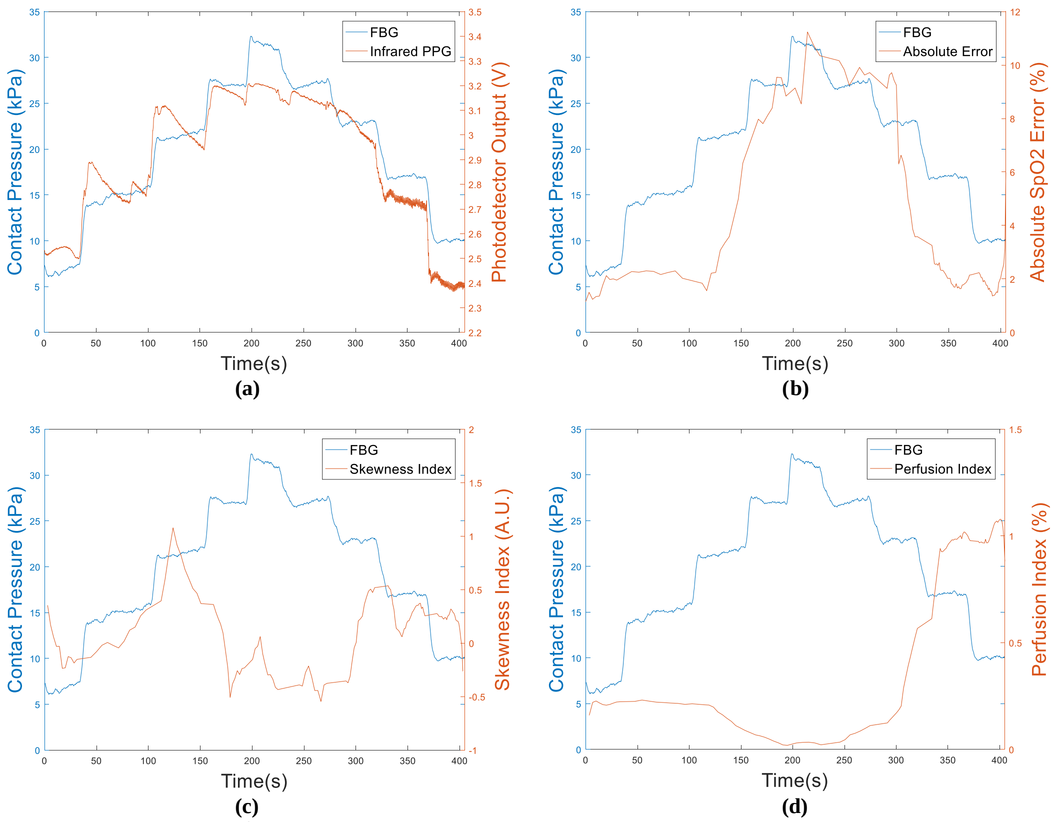

S 25. Volunteer 7-2. (a) FBG VS. Infrared PPG signals. (b) FBG VS. Absolute S_PO_2 error. (c) FBG VS. Skewness index. (d) FBG VS. Perfusion index.

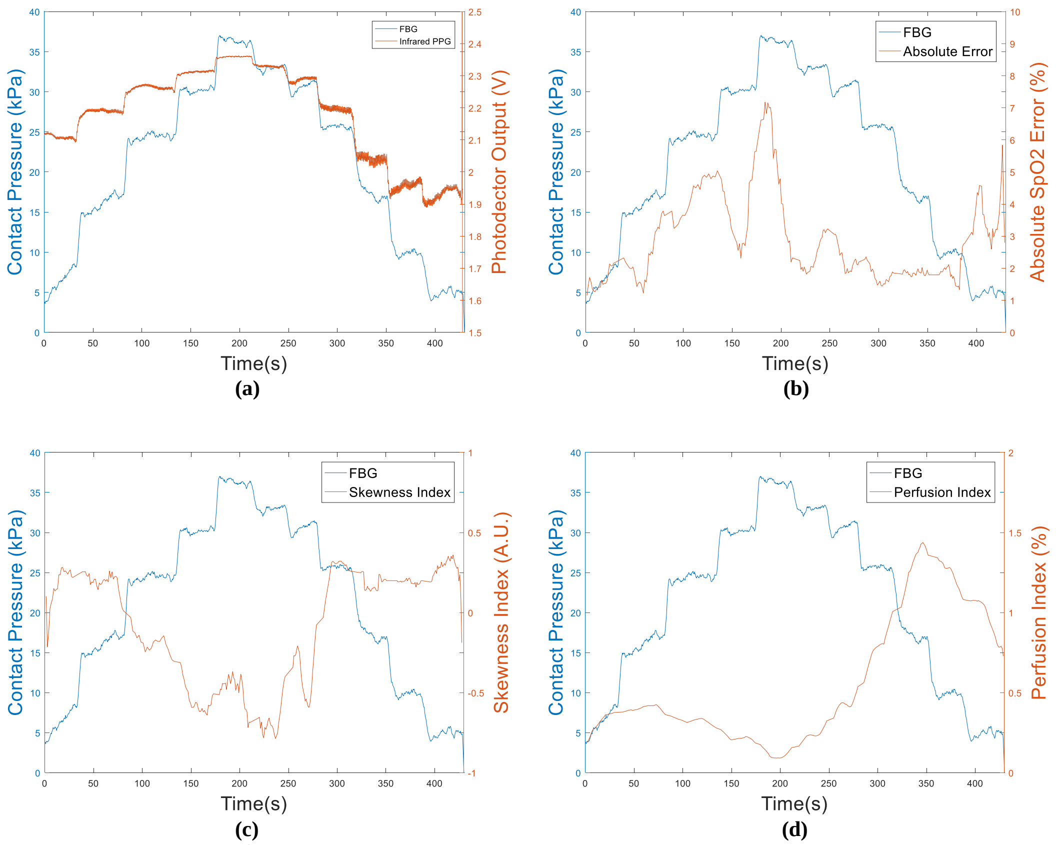

S 26. Volunteer 7-3. (a) FBG VS. Infrared PPG signals. (b) FBG VS. Absolute S_PO_2 error. (c) FBG VS. Skewness index. (d) FBG VS. Perfusion index.

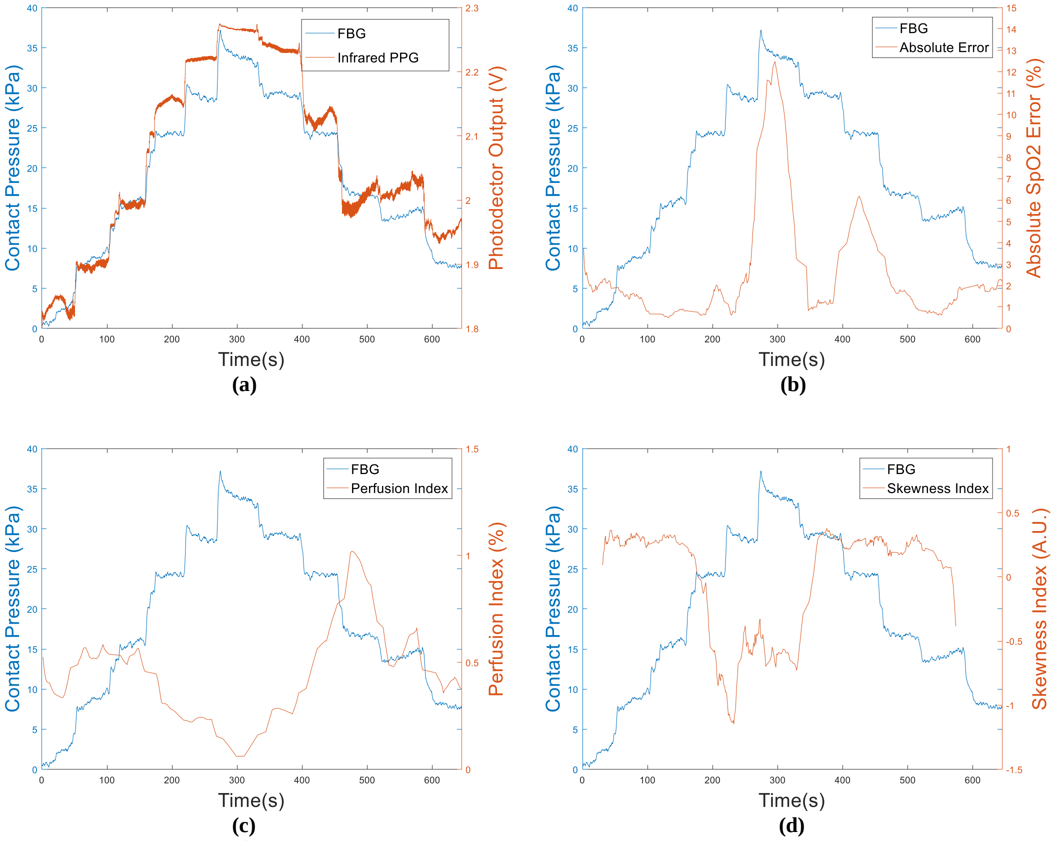

S 27. Volunteer 8-1. (a) FBG VS. Infrared PPG signals. (b) FBG VS. Absolute S_PO_2 error. (c) FBG VS. Skewness index. (d) FBG VS. Perfusion index.

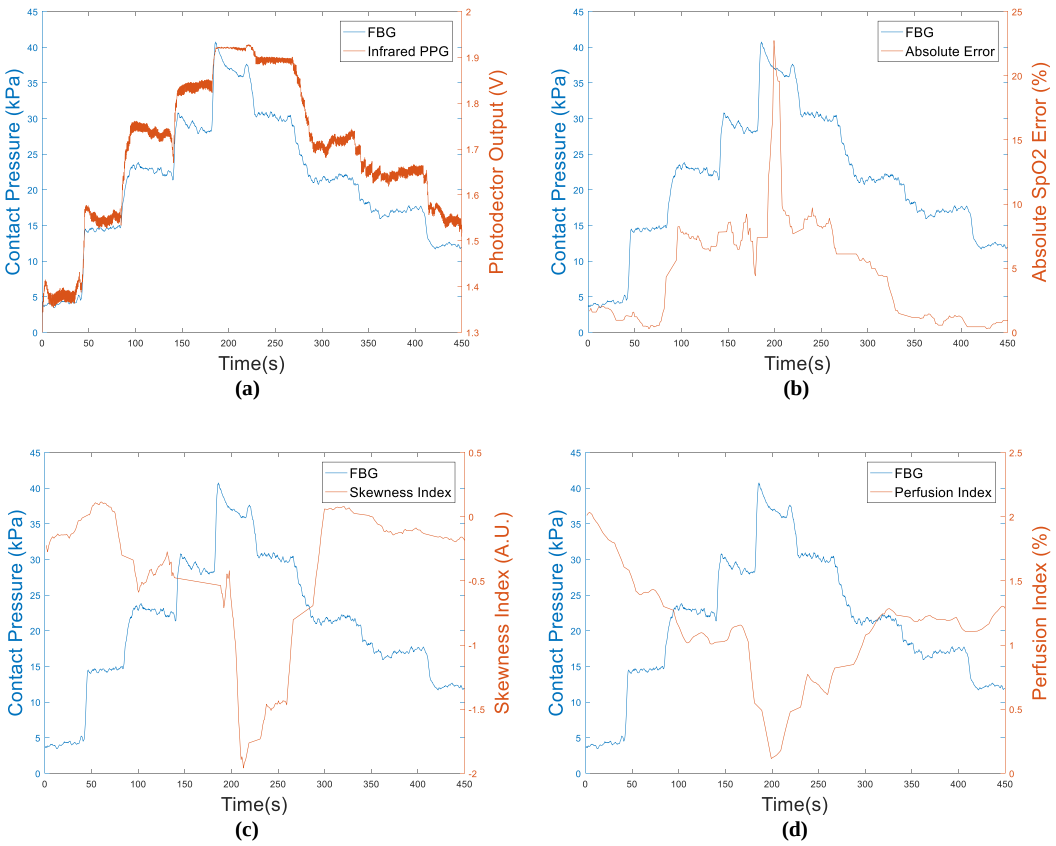

S 28. Volunteer 8-2. (a) FBG VS. Infrared PPG signals. (b) FBG VS. Absolute S_PO_2 error. (c) FBG VS. Skewness index. (d) FBG VS. Perfusion index.

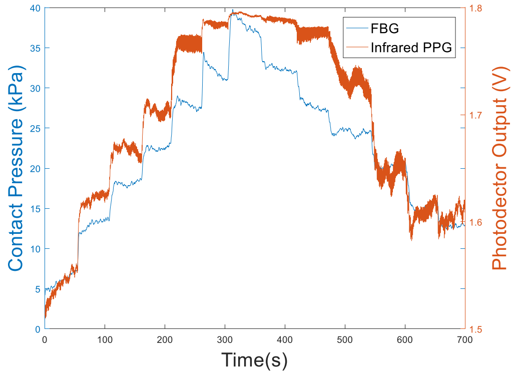

(a)

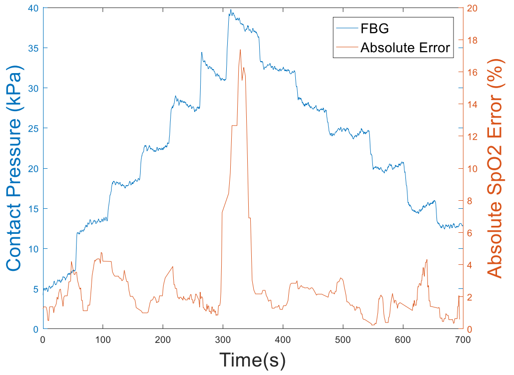

(b)

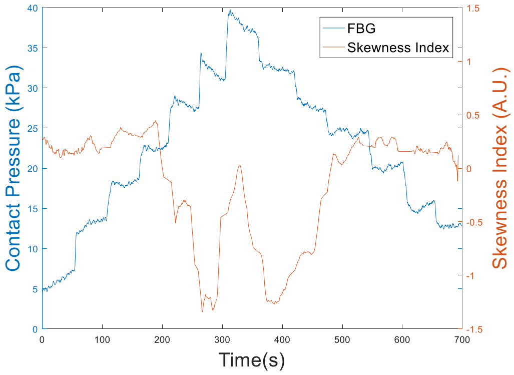

(c)

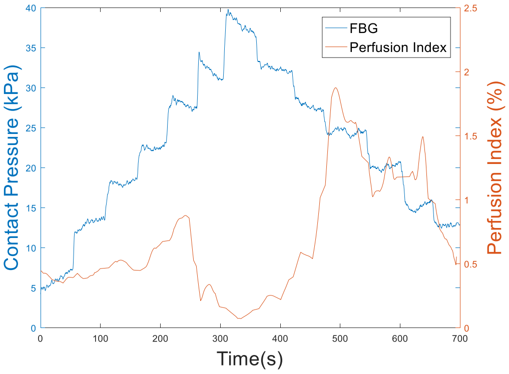

(d)

S 29. Volunteer 8-3. (a) FBG VS. Infrared PPG signals. (b) FBG VS. Absolute S_PO_2 error. (c) FBG VS. Skewness index. (d) FBG VS. Perfusion index.

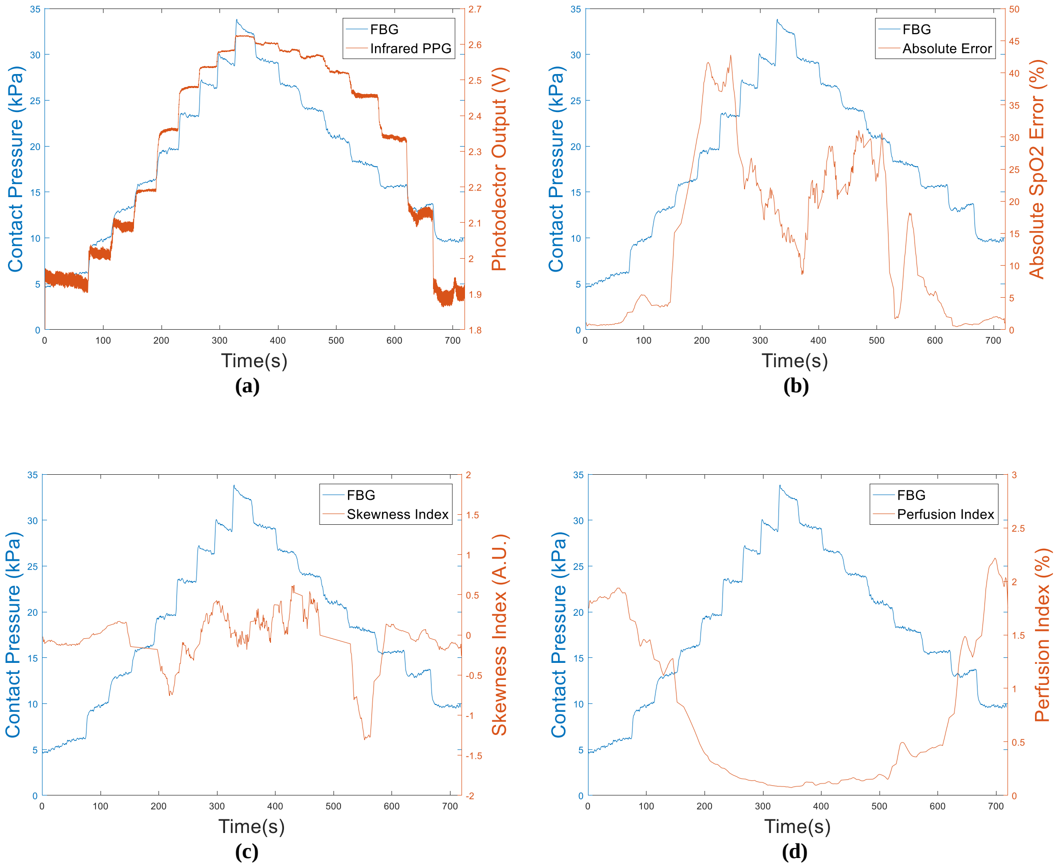

S 30. Volunteer 9-1. (a) FBG VS. Infrared PPG signals. (b) FBG VS. Absolute S_PO_2 error. (c) FBG VS. Skewness index. (d) FBG VS. Perfusion index.

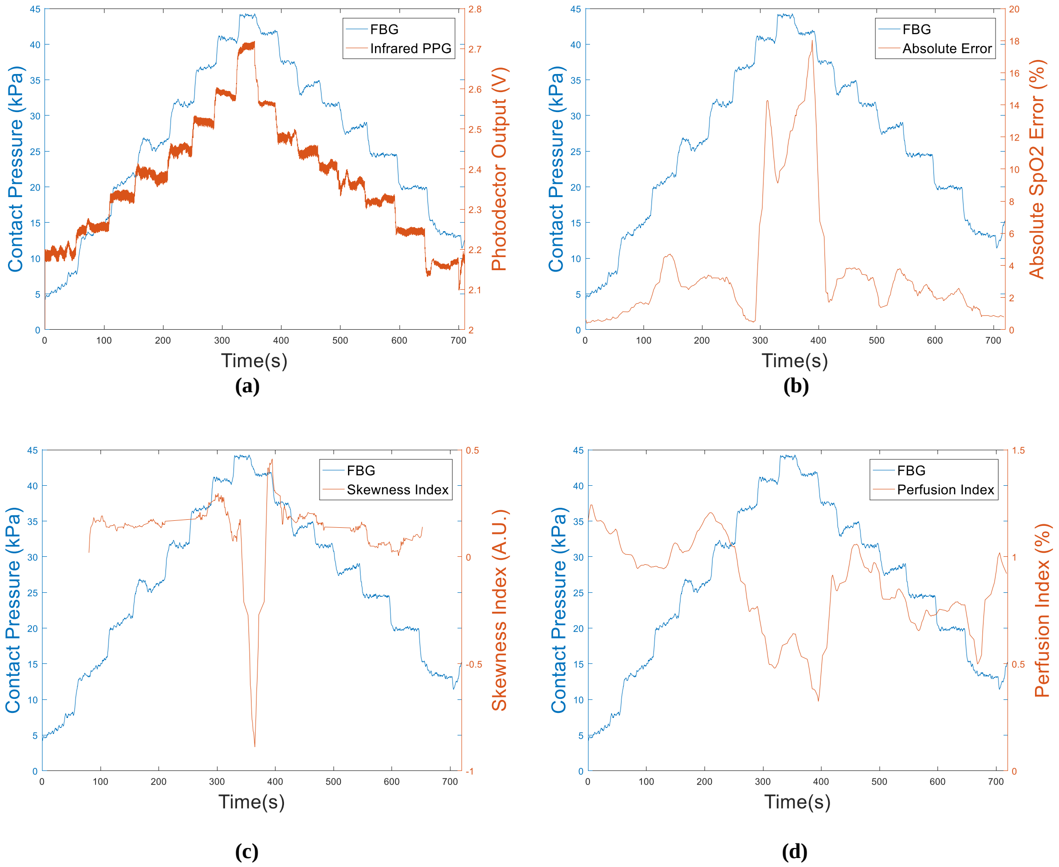

S 31. Volunteer 9-2. (a) FBG VS. Infrared PPG signals. (b) FBG VS. Absolute S_PO_2 error. (c) FBG VS. Skewness index. (d) FBG VS. Perfusion index.

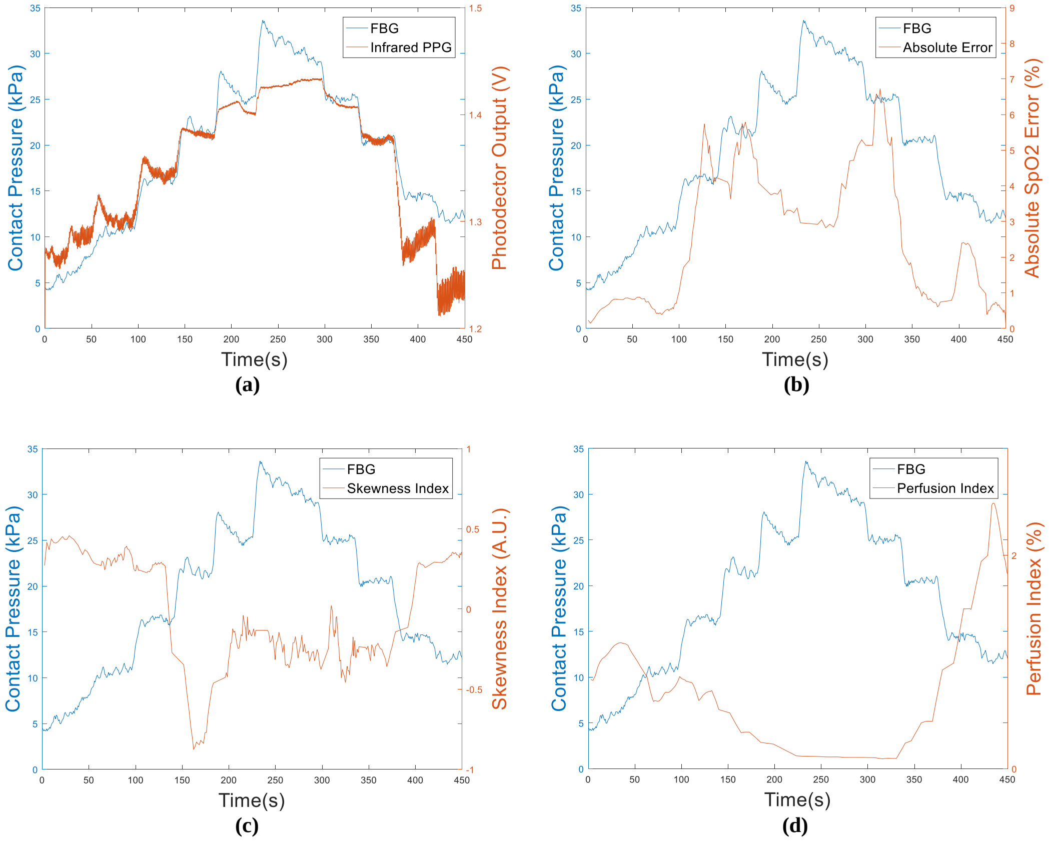

S 32. Volunteer 9-3. (a) FBG VS. Infrared PPG signals. (b) FBG VS. Absolute S_PO_2 error. (c) FBG VS. Skewness index. (d) FBG VS. Perfusion index.

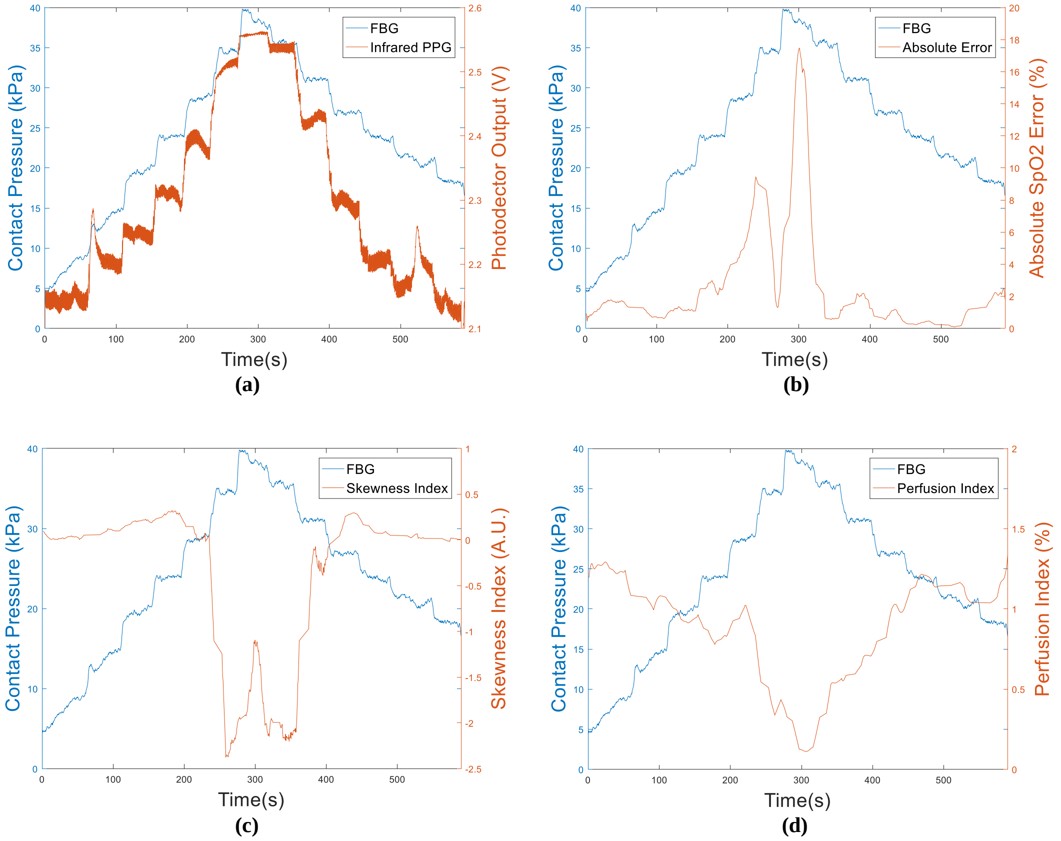

S 33. Volunteer 10-1. (a) FBG VS. Infrared PPG signals. (b) FBG VS. Absolute S_PO_2 error. (c) FBG VS. Skewness index. (d) FBG VS. Perfusion index.

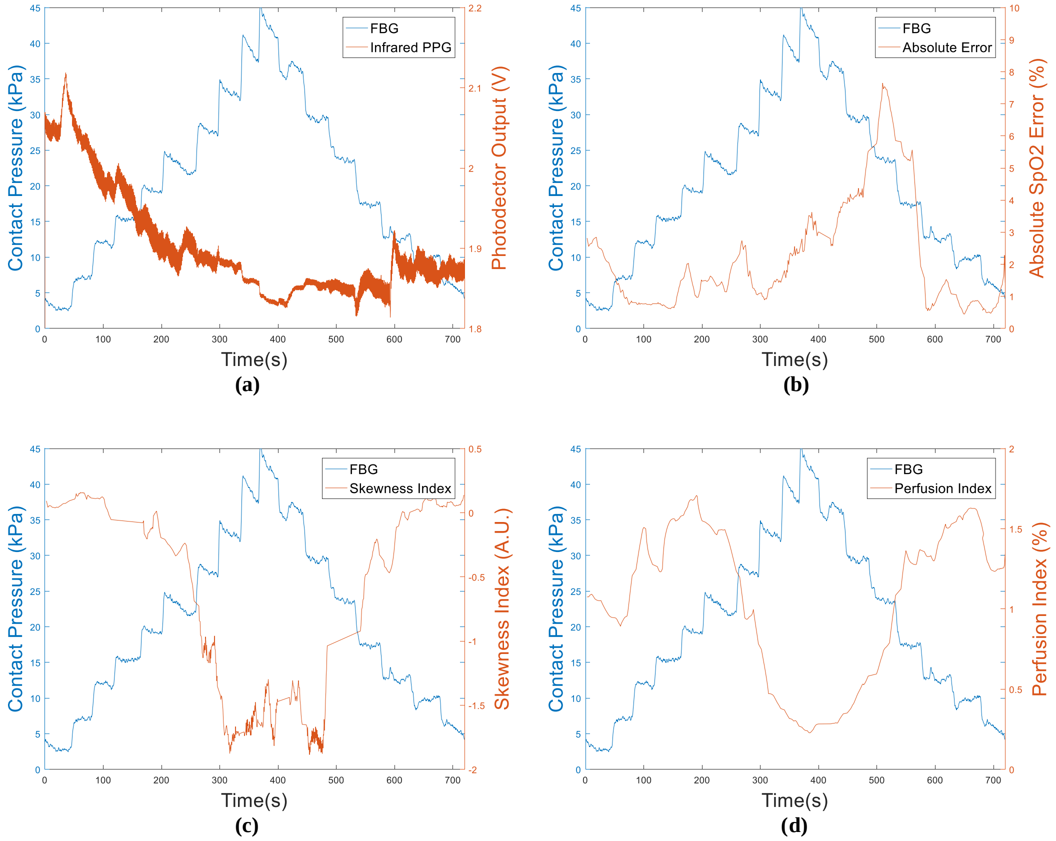

S 34. Volunteer 10-2. (a) FBG VS. Infrared PPG signals. (b) FBG VS. Absolute S_PO_2 error. (c) FBG VS. Skewness index. (d) FBG VS. Perfusion index.

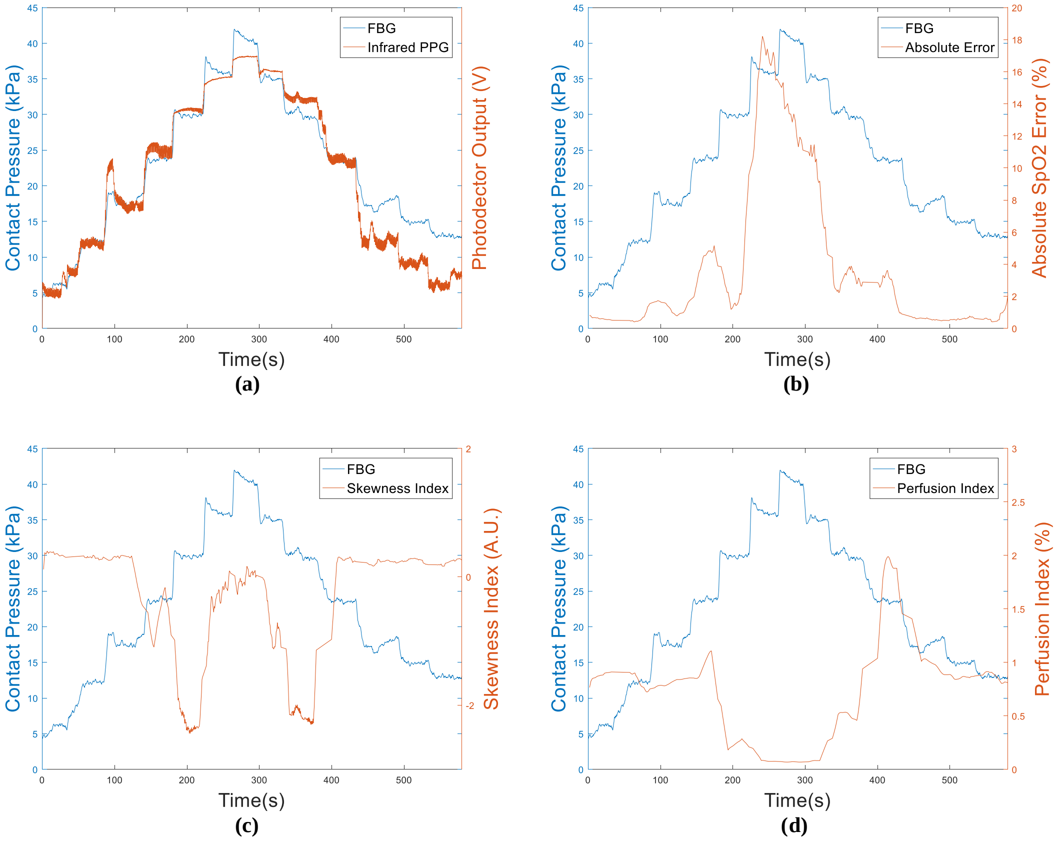

S 35. Volunteer 10-3. (a) FBG VS. Infrared PPG signals. (b) FBG VS. Absolute S_PO_2 error. (c) FBG VS. Skewness index. (d) FBG VS. Perfusion index.
